# Integration of democratic values in natural sciences education: A Literature Review of the last 50 Years

**DOI:** 10.12688/f1000research.154069.1

**Published:** 2024-08-20

**Authors:** Santiago Monsalve-Silva, Gabriel Otalvaro-García, Laura Sofía Cajica Velandia, Ana Dolores Vargas Sánchez

**Affiliations:** 1 Faculty of Education, Universidad de La Sabana, Chía, Cundinamarca, Colombia; 2 Faculty of Engineering, Universidad de La Sabana, Chia, Cundinamarca, Colombia

**Keywords:** education, democratic values, natural sciences, civil education

## Abstract

This study examines how democratic values have been promoted through natural sciences education over the last 50 years, providing a comprehensive analysis based on a systematic review of relevant literature. The central problem addressed is understanding the role of natural science education in fostering democratic values such as equity, participation, critical thinking, and ethical responsibility. This research aims to identify and analyze strategies, methodologies, and transformative experiences that contribute to the promotion of democratic values. This study employs the PRISMA methodology to ensure a rigorous and structured systematic review. Data were collected from multiple databases using detailed Boolean equations. Tools such as ScientoPy and VOSviewer were used for data preprocessing, clustering, and network visualization, followed by qualitative analysis to categorize the findings. Educational programs in natural sciences have increasingly integrated democratic values, fostering a culture of inclusivity and participation; the incorporation of ICT has enhanced equity and participation, while civic education has been fundamental in developing critical and informed citizens; citizen science initiatives have empowered students to engage in democratic deliberation and address epistemic injustice; cooperative learning methods in science classes have effectively promoted gender equity and inclusion; and emphasis on sustainability and environmental justice in science education has promoted democratic values and empowered students to take action on global challenges. In conclusion, natural science education is an effective vehicle for promoting democratic values, but it is an understudied field. By integrating practices that emphasize inclusivity, critical thinking, and ethical responsibility, science education not only enhances students’ scientific understanding but also prepares them to be active, informed, and responsible citizens.

## Introduction

Colombia and the world have faced historical crises due to armed conflicts, development processes, and the influence of global ideologies. Events such as the Second World War, the Cold War, and the Latin American dictatorships stand out, whose effects have left a mark on generations’ thinking and behavior. However, despite their negative impact, these events are fundamental for developing a reflective, scientific, and proactive society. Hence, an urgent need arises to cultivate a culture of peace that encompasses various dimensions of social coexistence, environmental preservation, personal growth, and interactions between individuals and knowledge. ^1^

The United Nations (UN), through UNESCO, has proposed recommendations to achieve world peace and contribute to human development through education. ^2^ It promotes comprehensive education that transcends educational standards and is applied in all contexts and levels, including rural, urban, conventional, and nonconventional areas, targeting people of all ages and social classes. ^3^

In its 42nd General Conference meeting, UNESCO ^3^ declared a series of aspects aimed at facilitating and promoting education for a culture of peace in all contexts. These aspects are based on current challenges in environmental, social, scientific, and technological areas.

In the 21st century, peace is redefined as an active process beyond conflict avoidance, emphasizing the appreciation of human dignity and mutual care for ourselves, others, and the planet. Educational systems play a crucial role in fostering this new understanding. They must equip individuals with the knowledge and skills to address climate crises sustainably, promote global citizenship through cooperation and dialog, and advance gender equality by ensuring equal access to education. In addition, in the digital age, media and digital literacy are paramount, enabling informed decision-making, critical thinking, and ethical online behavior. ^3^

Cabezudo and Haavelsrud ^4^ established that to perform peace education processes, it is essential to start with a series of fundamental ideas. These include understanding the meaning of “peace,” the role of the community in its construction, and the expected impacts in the short, medium, and long term. Progress has been made in defining the concept of peace, moving away from the traditional idea of simply “the absence of violence and the presence of social justice, participation, and diversity” (p. 10). Peace is understood as a process of freedom that recognizes the learning capacity of each individual and the importance of the sociocultural context in which they find themselves. ^5^ Dialog plays a crucial role in building this culture of peace, as it is both an act of human recognition and an ontological and epistemological necessity to seek truth together with others. Thus, it is recognized that building a peaceful society is not solely the responsibility of the social sciences, but all disciplines must address the challenges and issues raised, maintaining a close link between education for peace and a culture based on respect for human rights, non-violence, and dialog, thus promoting peaceful relationships and continuous transformation. ^6^

Therefore, education for peace is based on a series of pillars that facilitate the construction of a culture of peace. These pillars are known as the dimensions of education for a culture of peace. ^7^ In this case, we focus on the dimension of democratic values, as mentioned by Cerdas-Agüero. ^8^ Democratic exercise must always be accompanied by values and good attitudes, with the main ones being predisposition, conviction, and willingness to act coherently and responsibly. Likewise, ethical reflection and the expression of values are crucial for democratic life, especially in societies facing crises of civility and lack of positive values, which can lead to demoralization and confusion.

Given the above, it is crucial to recognize the strategies implemented in the last 50 years in peace education and its integration with current issues from the framework of sustainable development, taking into account international documents such as the UNESCO declaration on a culture of peace, whose central axis is the prioritization of a peaceful, integral, and sustainable society, providing a roadmap for the world and the achievement of the goals proposed in the document. Scientific disciplines play a fundamental role in promoting a culture of peace; therefore, this study analyzes the promotion of democratic values through the natural sciences using a scientometric methodology in databases covering the last 50 years. This is done to answer the following research question: “How have democratic values been promoted through the natural sciences in the last 50 years?”

## Methods

In this study, the PRISMA (Preferred Reporting Items for Systematic Reviews and Meta-Analyses) methodology was adopted to ensure a rigorous and structured systematic review. ^9^ The specific objectives of the study were initially defined to guide the selection and analysis of relevant literature. Subsequently, the inclusion and exclusion criteria for the studies were clearly established to focus on the most pertinent and high-quality publications. ^9^ For data collection, exhaustive searches were conducted across Scopus and Web of Science databases using a detailed search strategy with Boolean equations, as shown in Table 1.

**Table 1.** Boolean equations.

No.	Boolean equation
1	(“Science“ OR ”Natural Science“ OR ”Environmental education”) AND “democratic values”
2	(“Natural Science“ OR ”Environmental education”) AND “democratic values”
3	(“Natural Science“ OR ”Environmental education”) AND “learning” AND “democratic values”
4	(“STEM“ OR ”STEAM”) AND (“curriculum“ OR ”curricula“ OR ”education“ OR ”learning”) AND “democratic values”
5	(“Science“ OR ” Natural science“ OR ”Environmental education”) AND (“curriculum“ OR ”curricula“ OR ”learning”) AND “democratic values”

**Figure 1.**
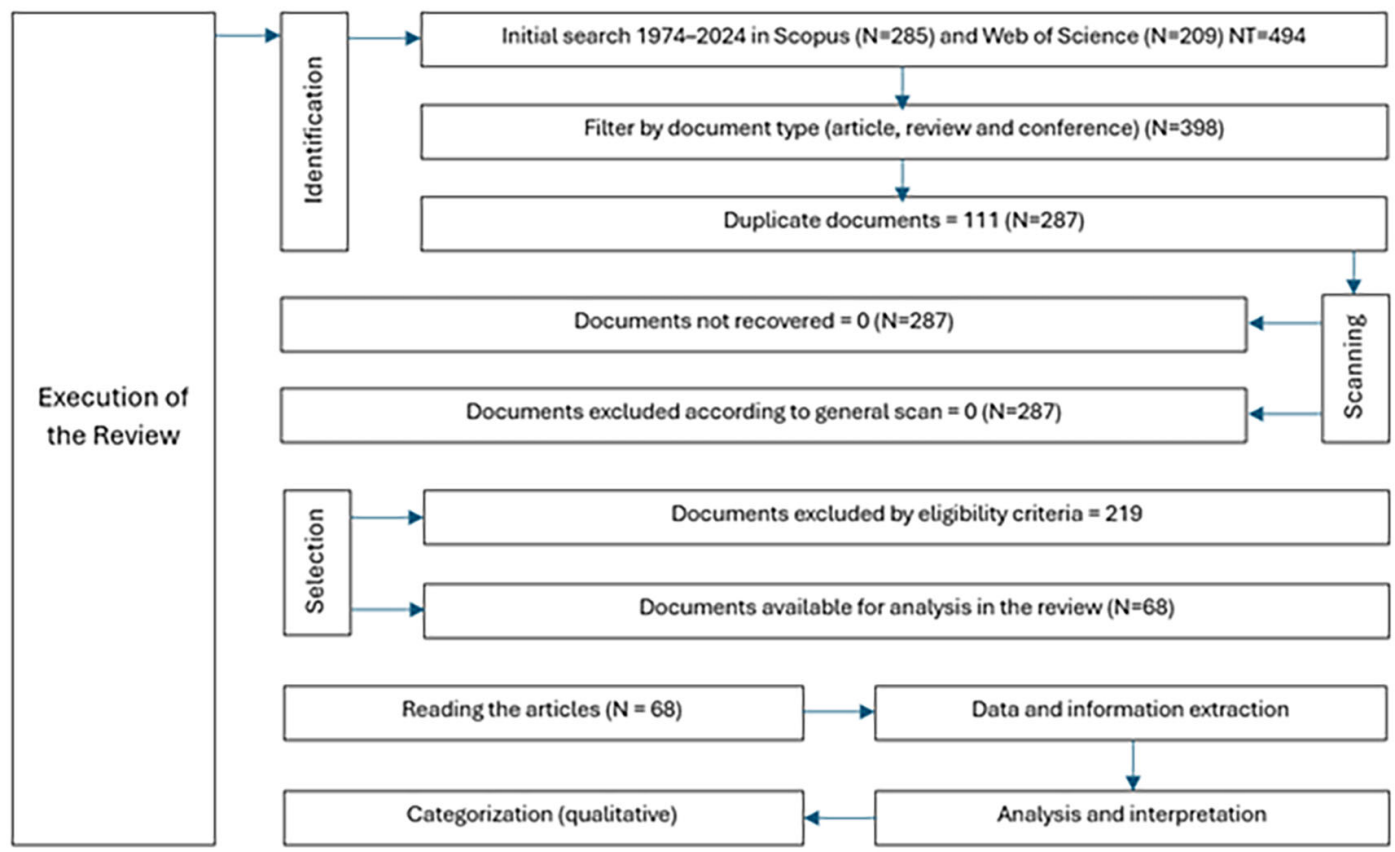
PRISMA flowchart for the literature review.

The study selection process was conducted using a two-stage approach, in which studies were initially filtered on the basis of titles and abstracts, followed by full-text evaluation for those that met the preliminary criteria: the relationship with democratic values and the relationship with education in natural sciences; the first filter focused on identifying studies that promote or evaluate the integration of democratic values such as equity, justice and citizen participation within the educational context, and the second filter addressed the specific connection with natural sciences education, seeking research that explores effective methodologies, content, and pedagogical practices in natural sciences teaching. This process is illustrated in a PRISMA flowchart ( Figure 1) that reflects the stages of selection and the reasons for study exclusion. Three reviewers performed a quick reading of the articles, focusing on summary, objective, methodology and results, they worked independently and then a second review was carried out analyzing the same criteria. Data extraction was performed independently by three reviewers to avoid bias, and any disagreements were resolved through discussion.

The extracted data were synthesized using tools such as ScientoPy ^®^ version 2.1.3 and VOSviewer ^®^ version 1.6.20 for preprocessing, subsequently facilitating the identification of clusters and interactions among the contents and authors. Finally, a qualitative analysis was conducted where six categories were defined, with each article being classified into a category, thus providing a comprehensive and systematic assessment of the existing literature, yielding well-founded conclusions.version 1.6.20 for preprocessing, subsequently facilitating the identification of clusters and interactions among the contents and authors. Finally, a qualitative analysis was conducted where six categories were defined, with each article being classified into a category, thus providing a comprehensive and systematic assessment of the existing literature, yielding well-founded conclusions.

To ensure the methodological rigor of this study under the PRISMA guidelines, the methods used to assess the risk of bias in the included studies were detailed thoroughly. Multiple independent reviewers were assigned to each study to ensure objectivity, and in cases of discrepancies, a third reviewer was consulted. Automated tools were utilized when necessary, particularly in the analysis and construction of clusters.

Regarding the synthesis methods, data extraction processes were employed, identifying primary outcomes, categorizing results, and deciding the eligibility of studies for each synthesis. This included tabulating the characteristics of each article and comparing them with the planned categories.

In terms of data preparation for presentation or synthesis, all articles were downloaded and systematically organized in MS Excel, where the analysis and categorization processes were conducted. For tabulating and visualizing results, the previously described tools were used to present the results of individual studies and syntheses. Sensitivity analyses were conducted, and possible causes of heterogeneity among the results were explored. For assessing the risk of bias due to missing results, a comprehensive reading of the findings with artificial intelligence was performed to confirm the identified ideas.

The data from the literature review process, integration of democratic values in natural sciences education, the PRISMA checkboard and the process flow diagram have been published by Ref. 10 and are located in the availability section.

## Results

According to the methodology outlined above, the following section presents an overview of the results obtained from the preprocessing, the networks constructed with the selected articles, and the findings from each category.

### Current trends identified

**Figure 2.**
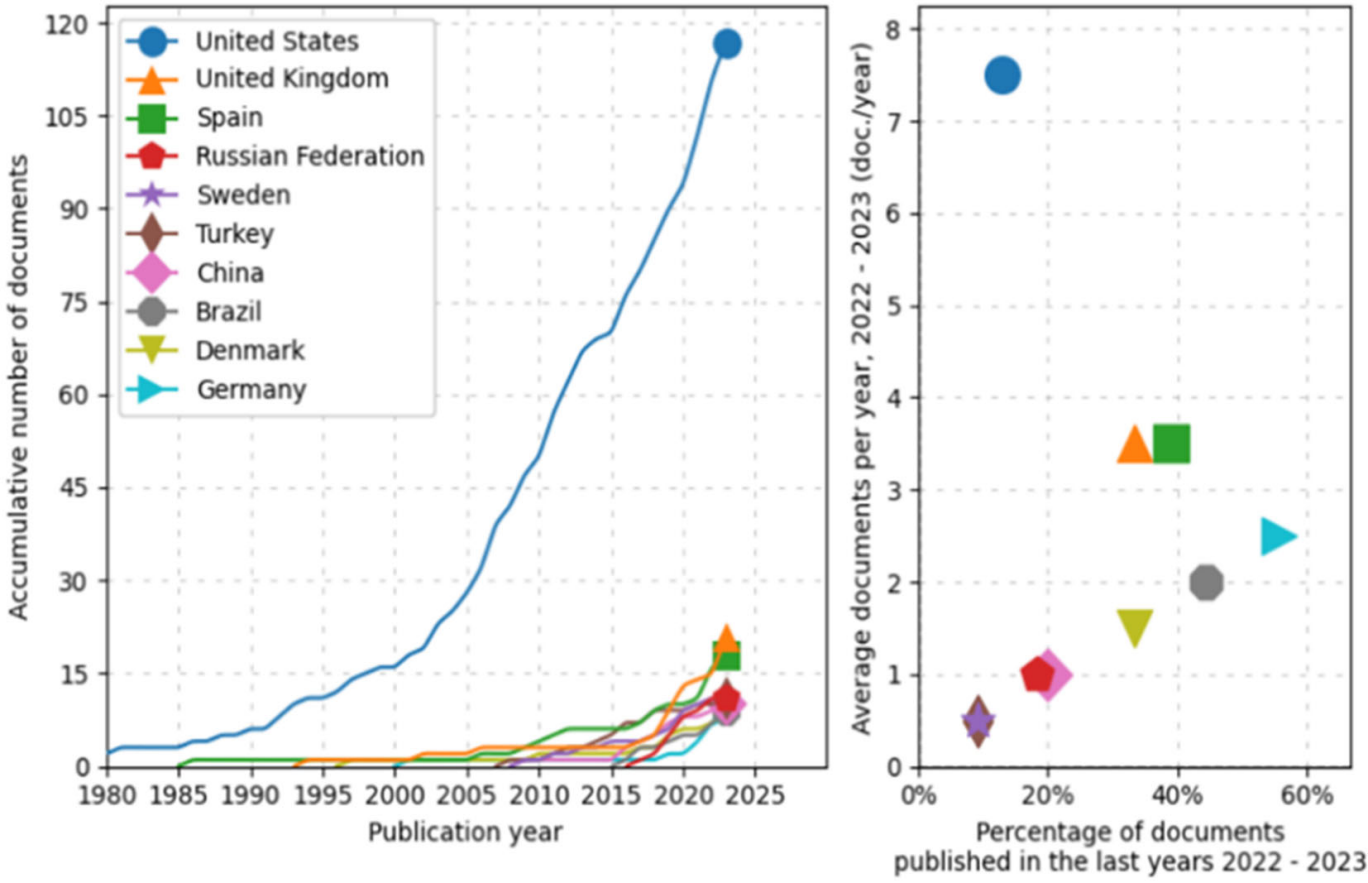
Scientific production. Source: the authors, made in ScientoPy.

First, during preprocessing, the evolution and distribution of scientific production from various countries over time is identified, as shown in Figure 2. The line graph on the left shows an exponential growth in scientific document production from the United States, particularly noticeable since 2000. In comparison, other countries such as the United Kingdom, Spain, the Russian Federation, Sweden, Turkey, China, Brazil, Denmark, and Germany also show an increase in the number of published documents, albeit more moderately. Notably, nations like China and Brazil have significantly intensified their document production in the last two decades.

The scatter plot on the right illustrates the recent activity (2022-2023) of these countries in terms of scientific publications. Although some countries show a lower percentage of recently published documents, the average number of documents published per year during this period is relatively high for some, a recent intensification in their research efforts. This analysis not only allows appreciation of the historical trajectory of scientific production from the selected countries but also helps identify current trends in their contributions to the global scientific corpus.

**Figure 3.**
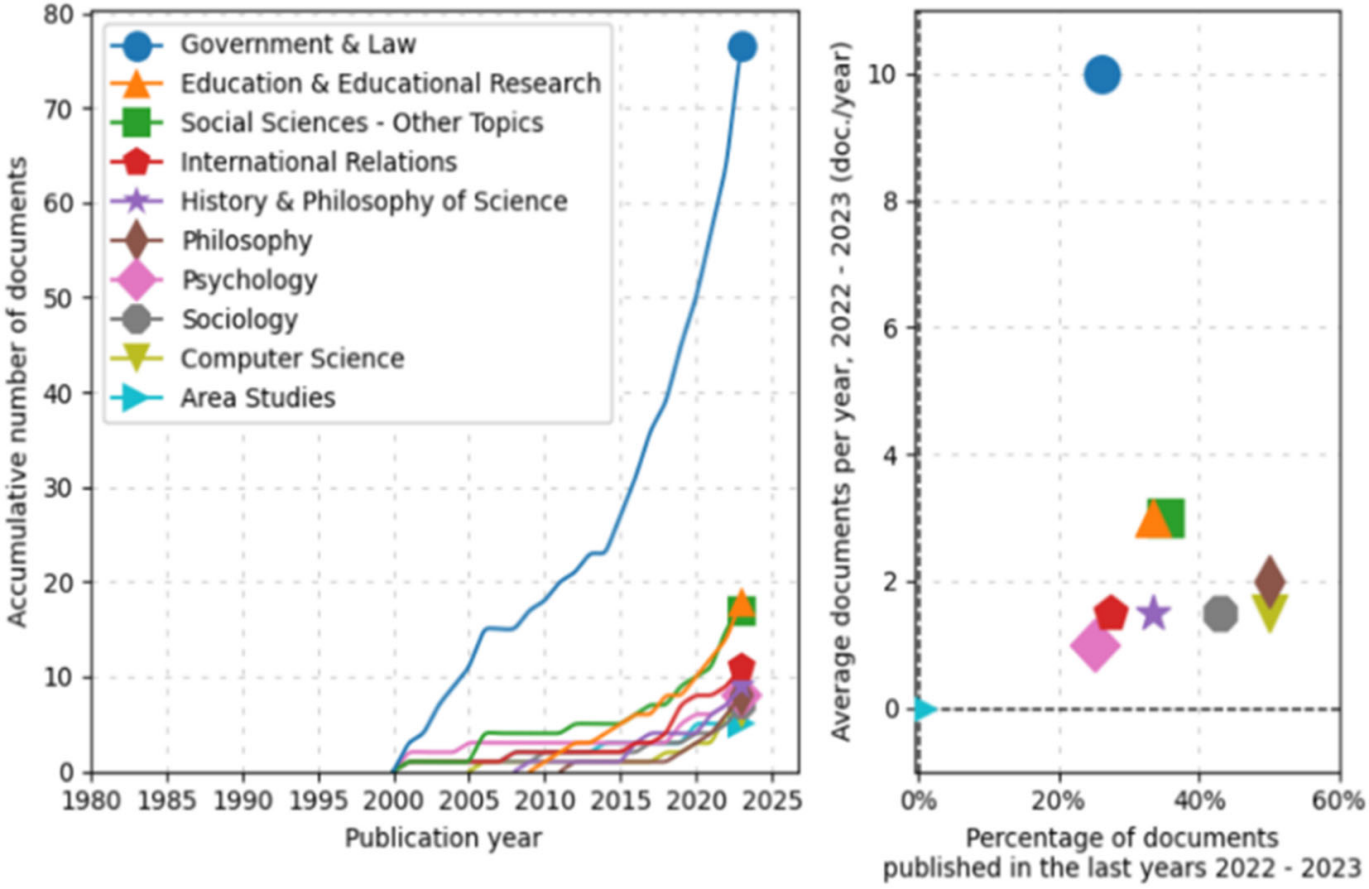
Trends by topic. Source: the authors, made in ScientoPy.

Subsequently, an analysis of trends by topic in documentary production from 1980 to 2025 was conducted, as shown in Figure 3. In the line graph, the field of “Government & Law” has experienced significant exponential growth, particularly starting in 2005 and continuing with a marked increase until 2025. Other fields such as “Education & Educational Research,” “Social Sciences-Other Topics,” and “International Relations” also show a steady increase, albeit less pronounced.

Regarding the scatter plot, which assesses the recent activity of these fields in 2022-2023, it can be seen that “Government & Law” not only dominates in terms of cumulative growth but also in recent annual average production, indicating a high ongoing activity in this field. Other fields, while having fewer cumulative documents, show a respectable average of recently published documents per year, a renewed or emerging interest in these topics.

**Figure 4.**
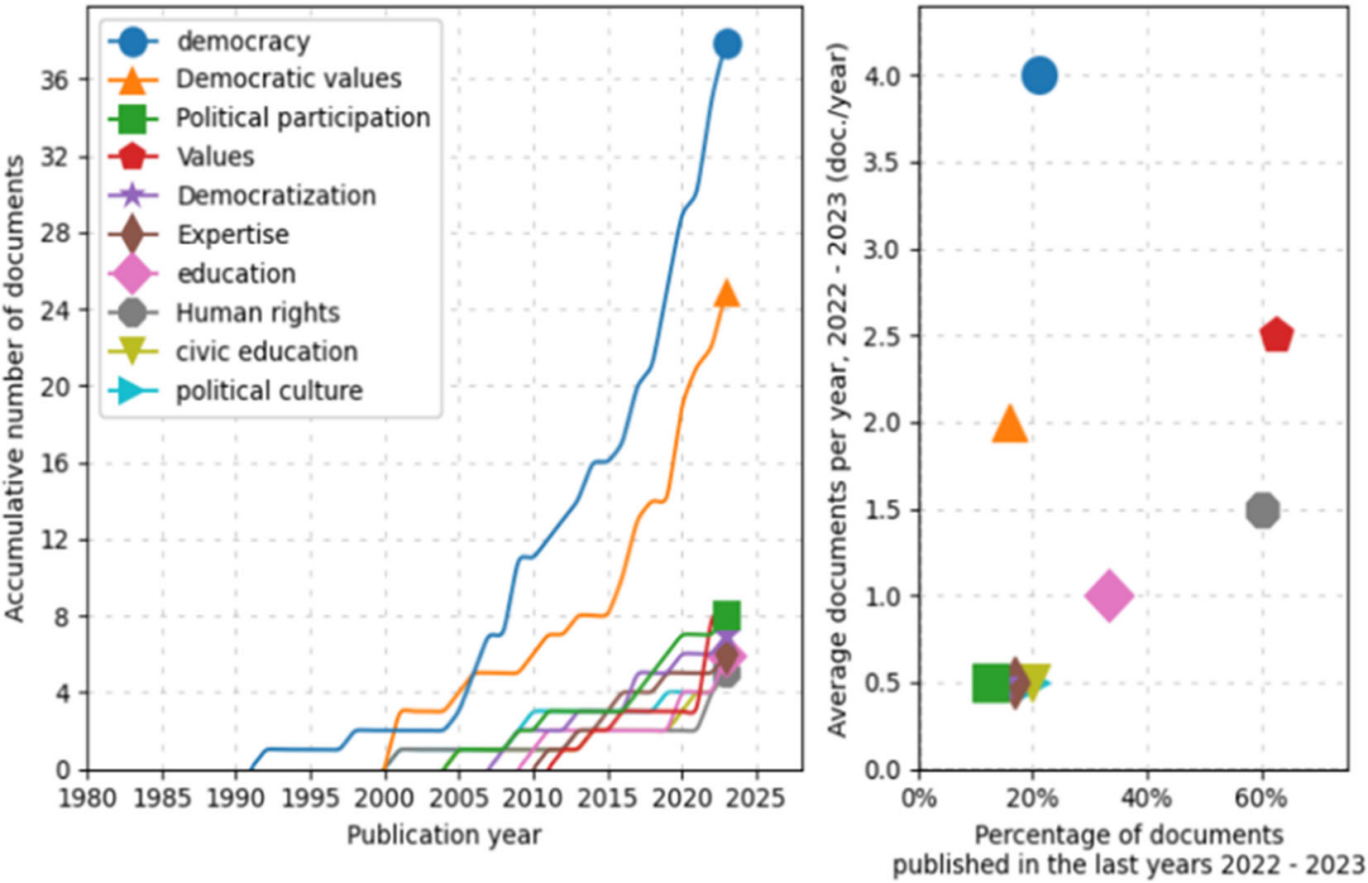
Trends by key words. Source: the authors, made in ScientoPy.

Similarly, a trend analysis using keywords was conducted, as shown in Figure 4. First, in the line graph on the left, the field of “democracy” shows a prominent exponential growth, especially after 2005, peaking in 2025. Other topics such as “Democratic values”, “Political participation”, and “Democratization” also exhibit a notable increase over time, although on a more moderate scale. Fields like “education” and “Human rights” demonstrate steady but less dramatic growth.

In the scatter plot on the right, which assesses recent publication activity (2022-2023) in these topics, it is evident that “democracy” not only leads in terms of cumulative growth but also maintains a high average of documents per year in the recent period, indicating a sustained and robust interest in studies on democracy. Topics such as “Democratic values” and “Political participation” show a significant percentage of recent documents, reflecting renewed focus on these themes in contemporary literature.

**Figure 5.**
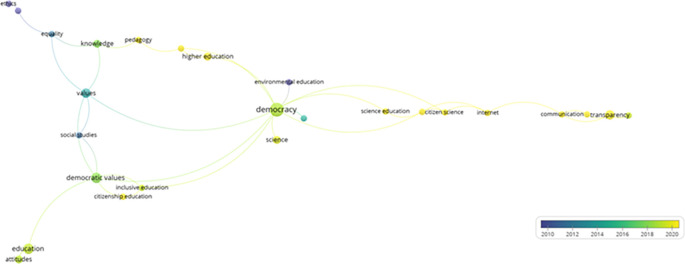
Key words network. Source: the authors, made in VosViewer.

Subsequently, from the selected articles that help to address the research question, a network of keywords was constructed in VOSviewer ( Figure 5), demonstrating how various themes related to education, democracy, and science are interconnected across seven main clusters.

The first cluster emphasizes citizenship education and encompasses aspects such as democratic values and inclusive education, highlighting the importance of integrating democratic principles into educational training to foster active social participation. The second cluster explores the relationship between science and citizenship, underscoring the role of the Internet in scientific dissemination and how it affects communication and transparency in the public sphere.

The third cluster links civics education with higher education and knowledge, proposing a synergy between civic training and advanced pedagogy. Meanwhile, the fourth cluster focuses on communication via social media, examining topics such as epistemic trust and transparency, and how these factors influence public perception and trust toward institutions. The fifth cluster connects democracy with environmental education and evolutionary biology, illustrating how educational approaches in science can complement and enhance understanding and participation in democratic practices, especially in the context of sustainability.

The sixth cluster addresses ethics, equality, and learning, underlining the relevance of these values in education and their impact on social and cultural development. Finally, the seventh cluster focuses on attitudes and education, exploring how predispositions toward learning and teaching affect educational processes.

This network provides a comprehensive view of how the interaction between these themes can generate new perspectives and opportunities for integrating the concepts of democracy, science, and education. It also highlights the importance of interdisciplinarity in the study of education and democracy, showing how different fields can collaborate to promote a more informed and participative society.

**Figure 6.**
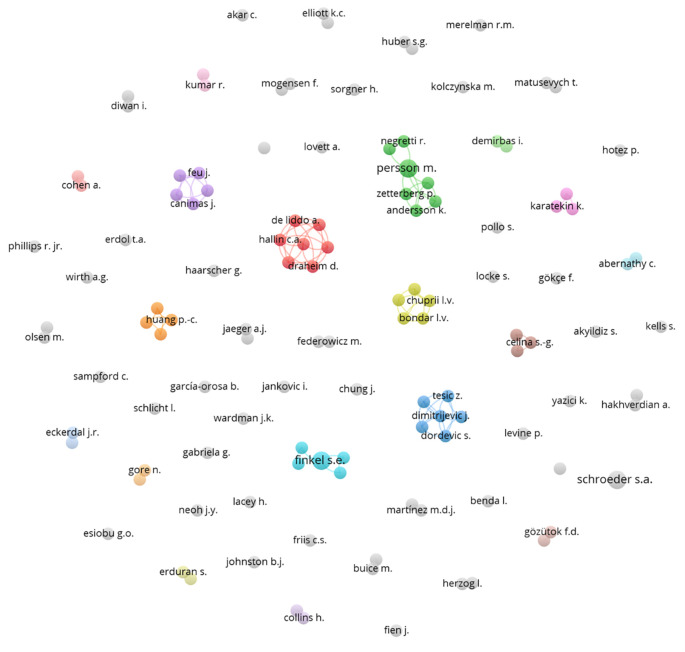
Authors network. Source: the authors, made in VosViewer.

A network of collaborations among various authors was subsequently constructed ( Figure 6), represented by several colored nodes. These nodes indicate groups of authors who frequently co-publish or are thematically related. Each node in the diagram represents an author, and the lines connecting the nodes indicate collaborations between them. The different colors assigned to the nodes may represent different fields of study, institutions, or simply co-authorship groups that have collaborated closely on several research projects.

The network shows low collaboration among various researchers, indicating a limited shared interest in integrating democratic values into science education. This organization suggests a multidisciplinary approach, where different aspects of science and democracy are addressed from various angles without effective integration.

The red cluster shows a group of authors who collaborate closely with each other, suggesting a cohesive approach and possibly a joint project or a series of publications related to specific topics within the study of democracy and natural sciences. Other clusters that demonstrate collaboration are the green, light blue, and orange clusters.

Each study was independently reviewed by two reviewers, and in cases of discrepancy, a third reviewer intervened to reach a consensus. The risk of bias assessment focused on aspects such as category selection, result extraction, outcome assessor blinding, and the handling of incomplete data. For the synthesis of the studies, key characteristics such as study design, sample size, educational context, and main outcomes were analyzed. The included studies varied in terms of methodological approach. Most studies showed a low risk of bias in critical areas.

Possible causes of heterogeneity among study results were investigated through collaborator network analysis and cluster analysis with keywords. Differences in contexts and methodologies used were the main sources of heterogeneity. Additionally, studies that used qualitative versus quantitative approaches showed heterogeneous results in terms of effectiveness and perception of democratic values.

### Development of teaching strategies, methodologies, and techniques

In the first instance, this category aims to classify articles that discuss and/or propose innovative approaches to teaching natural sciences in conjunction with democratic values, including innovative pedagogical methods and the integration of technologies in the classroom through interdisciplinary approaches. Initially, educational theory and philosophy were considered as the academic foundation for the relationship between education and politics, portraying the teacher and their methodologies as symbols of empowerment and social change, allowing for a series of criticisms and innovations in the educational systems of countries around the world, prioritizing students and their role in society. ^11^ The development of these methodologies has progressed as education was initially conceived as an alternative for social development (which is not wrong, but nowadays it is not limited solely to this field), incorporating democratic values into the school curriculum ^12^; Likewise, these types of models have been reconsidered over time, demonstrating how education provides the necessary tools to develop a democratic reflection exercise, thus promoting critical and thoughtful citizenship by integrating all disciplines, in this case, the natural sciences. Erduran and Kaya ^13^ proposed a series of questions for science teachers to promote scientific skills to achieve the goal of a critical and integral society regarding democratic values and how it can be achieved through various pedagogical models proposed in the research.

It is also important to recognize what Plaza De La Hoz ^14^ proposed, whose investigation delves into the evolving dynamic between educational authority and democratic values considering the integration of Information and Communication Technologies (ICT). Its dual objective is to analyze recent scholarly works concerning the impact of ICT on pedagogical authority and to corroborate these insights through empirical observation. Contrary to prevalent critical perspectives, the investigation posits that ICT serves to bolster teacher authority, albeit in a transformed manner, transitioning from a predominantly authoritative stance to one that is more demonstrative. Thus, corroborating the way in which not only education but also its approaches, strategies, and didactics have been advancing alongside political and social development.

### Transformative experiences in the promotion of democratic values in natural sciences

Contrary to what has been exposed, but to great surprise, methodologies with a positive impact have not stood out according to this study, since only one investigation belonging to Esiobu ^15^ has been found. This study investigates the impact of cooperative learning on promoting peace, equality, and equity in science classrooms, which are essential for sustainable development within a democratic framework. Through a gender equity and peace questionnaire, we found that cooperative learning effectively fosters equity and peace among genders in the biology classroom. This establishes a strong relationship between the Sustainable Development Goals and the development of scientific thinking in the classroom, thus promoting democratic values within the institution.

### Presence of democratic values in scientific formal education programs

The integration of science into educational programs has become pivotal for developing scientific skills within communities, particularly in areas concerning the environment and community health. Through cienciometric tracking, a wide array of documents emphasizing the significance of promoting environmental education for ecosystem conservation and fostering democratic values for sustainable development have been identified. ^16^ However, this challenge is not confined to classrooms; it also poses a dilemma for policymakers, as environmental governance is essential for devising strategies that safeguard the environment and biodiversity through effective governance. ^17^

Presently, environmental education is on the rise, with natural sciences focusing on sustainable applications and explanations of environmental phenomena. This interdisciplinary field permeates all areas of knowledge because of its versatility and is closely linked to democracy and its values. Terms such as democracy and environmental justice are addressed, empowering educators, students, and policymakers to reconsider their impact on preserving the world. ^18^ Environmental education is incomplete without action, as highlighted by Mogensen and Schnack, ^19^ who underscore the crucial role of action in sustainable development processes in society. Since 1980, this component has been integrated into Denmark’s educational programs, resulting in significant shifts in population dynamics and bolstering democratic values regarding the environment across all knowledge domains.

Despite science being fundamental for societal development, uncertainties persist regarding sustainable development, biodiversity conservation, and the contribution of democratic values to achieving the Sustainable Development Goals. ^20^ The emergence of new technologies offers opportunities for scientific and social innovation, prompting educational and governmental institutions to develop action plans to consolidate scientific knowledge within communities. It is imperative to not overlook the importance of democratic values in ensuring global sustainable development ^21^^,^^22^

### Democracy: a journey

The importance of integrating democratic values into various fields of knowledge has been emphasized; however, it is crucial to specify which types of democratic values should be promoted through different research. Initially, these values must be enhanced through active participation; nevertheless, highlighted challenges in student motivation, fewer female candidates and representatives, and gender biases favoring male candidates have been identified. Female students faced obstacles and lacked democratic campaign methods; however, they challenged gender norms by supporting female candidates. ^23^

Similarly, new technologies pose a fresh challenge to democratic values. The emergence of digital disruptions, particularly facilitated by platforms like social media, presents a notable obstacle to democratic principles and collective wisdom. Concerns such as misinformation, political biases, and increasing distrust in institutions have the potential to undermine the efficacy of collaborative endeavors. Safeguarding the pivotal role of collective participation in confronting contemporary challenges requires immediate action to address issues like inclusivity, openness, and group dynamics. ^24^ Recent surges in antivaccine activism and other antiscience trends have converged with rising antisemitism. A clear example of this phenomenon arose during the COVID-19 pandemic, as far-right elements often employed Nazi imagery to criticize vaccinations and even blamed the Jewish people for COVID-19 and vaccine profiteering. These parallels with historical persecution underscore the urgent need for action. ^25^

Moreover, democratic values are linked to science in society. Initially, the focus was on reflecting on the relevance of scientific disciplines in democracy, and vice versa, integrating the foundations of both natural and social sciences, reaching scientific and democratic philosophy, and presenting points of disagreement or agreement. ^26^ In essence, both fields of knowledge are interdependent, considering that natural sciences provide explanations for phenomena that facilitate or challenge human development; likewise, social sciences provide knowledge regarding human behavior and society, relating aspects such as justice, equity, and recognition of various ethnicities and populations.

These sciences are at the forefront of exercising democratic values concerning the environment, gender, and sustainable development. ^27^ Thus, in democratic societies, the threat of populism persists, but science can help mitigate this risk. By upholding shared values like honesty and universalism, science serves as moral leadership, preventing the rise of populism. Additionally, science contributes to a system of checks and balances that limit executive power. However, an excess or deficiency of science can lead to democratic failures, highlighting the need to reaffirm democratic values. Meanwhile, a study on tenured academics’ experiences in science communication reveals their commitment to sharing knowledge beyond academia, despite the challenges it poses to their academic work. They engage in various writing practices driven by a desire to educate and democratize scientific knowledge. Further research and training programs are required to enhance science communication skills among future scientists. ^28^^–^^30^

### Civic education

Civic education seeks to promote critical awareness of social, political, and environmental issues, fostering respect for diversity and a commitment to the common good. As noted by Matusevych and Shevchuk, ^31^ civic education is a fundamental tool for building trust across various domains, with the aim of developing social responsibility grounded in ethical principles and the pursuit of the common good. This approach not only aims to inform students about their rights and duties as citizens but also motivates them to actively participate in society and contribute to its improvement.

Similarly, education for citizenship focuses on preparing citizens to navigate the dynamism of modern societies. According to Neoh, ^32^ it is crucial that curricula are designed to reflect the objectives and developmental needs of contemporary society. This necessitates a constant adaptation of educational content to ensure that students are well equipped to face contemporary challenges, such as globalization, digitalization, and environmental changes.

However, Feu et al., ^33^ underscored the importance of addressing key concepts in a unified manner within the educational sphere. According to this study, for the teaching-learning process to be effective, it is essential that all educational actors (teachers, students, administrators) share a common and cohesive understanding of the principles and objectives of civic education. This ensures that the values and knowledge imparted are coherent, facilitating their internalization and practical application by students.

### Teacher training

In this category, strategies for initial and ongoing teacher training are analyzed to prepare educators for classroom challenges by integrating democratic values such as equality, inclusion, and mutual respect into their pedagogical practice. Johnston ^34^ suggests that leadership, the pursuit of democratic values, and the structural and cultural dimensions of school organization are fundamental for continuous societal improvement. It is crucial to define the roles of formal school leaders, teachers, and other school staff to create an educational environment that supports democratic objectives.

Additionally, Yazici ^35^ presented a study on the democratic values of prospective teachers using the Democratic Teacher Values Scale. The study indicates that future teachers exhibit high levels of democratic values in terms of solidarity, the right to education, and freedom. This study underscores the need to incorporate these values into teacher training programs so that teachers can effectively promote a learning environment that reflects these principles. It concludes that factors such as the university attended and the father’s educational background significantly impact these values, highlighting the importance of a comprehensive and contextualized approach to teacher training.

Finally, Gökçe ^36^ and Dogru and Demirbas ^37^ explored teachers’ perceptions and expectations regarding the parameters of rights, freedoms, and responsibilities within democracy. Gökçe revealed a significant discrepancy between the observations and expectations of prospective teachers concerning these values, indicating an urgent need to align educational practices with democratic ideals. On the other hand, Dogru and Demirbas found a positive and moderate relationship between teachers’ perceptions of multicultural competence and their democratic values, suggesting that strengthening these values can enhance teachers’ ability to manage classroom diversity. Both studies emphasize the importance of teacher training that not only imparts knowledge but also fosters a culture of justice, respect for differences, and equity.

## Discussion

First, there is a clear convergence on the notion that education should transcend the mere transmission of technical knowledge, incorporating democratic values essential for the development of a more just and participatory society. The integration of ICT, civic education, and service-learning are prominent strategies that promote equity, participation, and the moral and intellectual development of students. Scientific argumentation and deliberative democracy are presented as complementary methods that enrich teaching and foster critical and participatory understanding among students.

Friis ^12^ and Plaza de la Hoz ^14^ highlight how ICT can enhance equity and participation in education, demonstratively reinforcing pedagogical authority. Abernathy and Forestal, ^38^ along with Iskhakova et al., ^39^ emphasize the need for civic and political education to cultivate informed and engaged citizens by employing both extracurricular events and innovative approaches to political education. Wirth ^40^ and Erduran and Kaya ^41^ explored the intersection between science and democracy, proposing that scientific education should include practices of argumentation and democratic deliberation to develop critical and participatory skills. Finally, Óscar, Celina, and Jesús ^42^ advocated service-learning as a methodology that combines academic learning with community service, fostering social responsibility and civic engagement.

Feu et al. ^43^ addressed the need for a precise and multifaceted understanding of democracy within the educational sphere, highlighting four dimensions: governance, habitance, alterity, and ethos. This framework can be applied to science education to promote an inclusive and participatory school culture, thereby fostering ethical responsibility and respect for diverse perspectives.

Thornton and Jaeger ^44^ complement this vision by emphasizing civic responsibility within higher education. They argue that promoting scientific knowledge and skills for societal benefit and appreciation for diversity are pertinent to science education, which can contribute to forming students aware of their societal roles.

Conversely, Purevdagva et al. ^45^ underscored the importance of fact checking and critical thinking in scientific education. Educating students about the significance of critically evaluating and understanding the world is essential for fostering informed and engaged citizenship.

Herzog and Lepenies ^46^ mention that citizen science can promote deliberative democracy and address epistemic injustice. Integrating active student participation in dialog about the direction and impact of scientific research encourages scientifically informed and engaged citizens.

Esiobu ^47^ demonstrated how cooperative learning in science classroom can promote gender equity and inclusion. By showing that this methodology can overcome gender barriers and promote equality, cooperative learning is essential for democratic education.

Additionally, Federowicz and Terepyshchyi ^48^ support this vision by highlighting how integrating democratic values into the Polish educational system can facilitate cultural understanding, civic engagement, and social inclusion. As Thornton and Jaeger ^44^ mention, civic responsibility within higher education is an approach applicable to natural sciences that promotes the use of scientific knowledge for societal benefit and fosters an appreciation for diversity.

However, Sorgner ^49^ discusses the relationship between scientific authority and democratic participation in decision making. He addresses the balance of expert knowledge with the inclusion of diverse perspectives and democratic values in scientific education to prepare students for engaging in critical dialogs about science and society.

Manias et al. ^50^ discussed the need for transparency and accountability in the use of AI in governance. They emphasize the importance of educating natural science students about ethical and democratic principles concerning technology, promoting a critical understanding of how technology can be ethically and responsibly designed and used.

As Schroeder ^51^ mentions, science should appeal to democratic values, meaning the values of the public or their representatives, to maintain or even strengthen public trust in scientific research. He emphasizes the importance of integrating democratic values into scientific education.

Thus, the integration of democratic values into education has significant implications for educational policies and pedagogical practices. To develop a more equitable and democratic society, it is essential to implement policies that promote equity and accessibility in education using innovative technologies and methodologies. Furthermore, fostering civic and political education from an early age is crucial for developing informed and engaged citizens.

In this way, the practices of democratic deliberation and argumentation in scientific education can enrich students’ understanding of the interconnection between science and democracy, preparing them to actively participate in scientific and political debates. Similarly, service-learning, as a pedagogical methodology, offers a practical approach to integrating academic learning with community service, promoting democratic values, and developing personal and professional competencies in students.

## Conclusions

The findings suggest that the promotion of democratic values through science is an under-researched area; however, it is inferred to be a multidimensional process that includes the incorporation of inclusive practices, the fostering of critical thinking, gender equity, ethical responsibility, and civic participation. These approaches not only enhance the quality of scientific education but also prepare students to become informed and engaged citizens in a democratic society.

Furthermore, the importance of developing curricula and educational policies that integrate democratic values into the teaching of natural sciences is emphasized. This not only improves scientific education but also strengthens democracy by preparing students to be active, critical, and responsible citizens.

The incorporation of information and communication technologies (ICT) has been a key element in promoting equity and participation in education. Friis and Plaza de la Hoz demonstrate how ICT can reinforce pedagogical authority and facilitate more inclusive and participatory education. Additionally, civic education, as noted by Purevdagva et al., is fundamental in forming informed and engaged citizens capable of critically evaluating information and participating in scientific and political debates.

Herzog and Lepenies emphasize the importance of integrating active student participation in scientific research, to foster a scientifically informed and engaged citizenry. This approach enables students to better understand the relationship between science and social values, thereby promoting greater involvement in scientific decision-making.

Finally, promoting civic and political education from an early age, as well as incorporating practices of democratic deliberation and argumentation in scientific education, can enrich students’ understanding of the interconnection between science and democracy.

### Ethics and consent

Ethical approval and consent were not required

## Data Availability

No data associated with this article. S. Monsalve-Silva, J. G., Otalvaro-García, L. S, Cajica Velandia, & A. D., Vargas Sánchez. Extended data Integration of democratic values in natural sciences education [Data set]. Zenodo. 2024. doi: https://doi.org/10.5281/zenodo.13229007. ^10^ The project contains the following extended data:
1.

Flow_diagram_Integration of democratic values in natural sciences education.png
2.
Literature Data Integration of Democratic Values.xlsx
3.

PRISMA_Checklist_Literature Data Integration of Democratic Values.docx Creative Commons Zero v1.0 Universal
