# Integration of Democratic Values ​​in Natural Sciences Education: a Review of the Literature of the Last 50 Years

**DOI:** 10.12688/f1000research.154069.4

**Published:** 2025-09-12

**Authors:** Santiago Monsalve-Silva, Gabriel Otalvaro-García, Laura Sofía Cajica Velandia, Ana Dolores Vargas Sánchez

**Affiliations:** 1 Faculty of Engineering, Universidad de La Sabana, Chia, Cundinamarca, Colombia; 2 Faculty of Education, Universidad de La Sabana, Chía, Cundinamarca, Colombia

**Keywords:** education, democratic values, natural sciences, civil education

## Abstract

This study examines how democratic values have been promoted through natural sciences education over the last 50 years, providing a comprehensive analysis based on a systematic review of relevant literature. The central problem addressed is understanding the role of natural science education in fostering democratic values such as equity, participation, critical thinking, and ethical responsibility. This research aims to identify and analyze strategies, methodologies, and transformative experiences that contribute to the promotion of democratic values. This study employs the PRISMA methodology to ensure a rigorous and structured systematic review. Data were collected from multiple databases using detailed Boolean equations. Tools such as ScientoPy and VOSviewer were used for data preprocessing, clustering, and network visualization, followed by qualitative analysis to categorize the findings. Educational programs in natural sciences have increasingly integrated democratic values, fostering a culture of inclusivity and participation; the incorporation of ICT has enhanced equity and participation, while civic education has been fundamental in developing critical and informed citizens; citizen science initiatives have empowered students to engage in democratic deliberation and address epistemic injustice; cooperative learning methods in science classes have effectively promoted gender equity and inclusion; and emphasis on sustainability and environmental justice in science education has promoted democratic values and empowered students to take action on global challenges. In conclusion, natural science education is an effective vehicle for promoting democratic values, but it is an understudied field. By integrating practices that emphasize inclusivity, critical thinking, and ethical responsibility, science education not only enhances students’ scientific understanding but also prepares them to be active, informed, and responsible citizens.

## Introduction

Colombia and the world have faced historical crises due to armed conflicts, development processes, and the influence of global ideologies. Notable events include the Second World War, the Cold War, and the Latin American dictatorships stand out, whose effects have left a mark on generations’ thinking and behavior. Such events have shaped the context, culture, customs, and traditions of Colombia society. Various authors have highligthed the presence of structural and cultural violence, in addition to direct violence. ^1^ These dynamics have also influenced citizen participation and the country’s democratic system, which has been affected by issues such as corruption and lack of transparency in governmental operation. ^2, 3^ Given this, there is an urgente need to address democratic values education more deeply across all fields of study.

Although the United Nations (UN), through UNESCO, has made recommendations to achieve world peace and contribute to human development through education, ^4^ as well as to promote comprehensive education and democracy that transcend educational standards and are applied across all contexts and levels, including rural, urban, conventional, and nonconventional settings, and aimed at people of all ages and social classes. ^5^ Colombian citizens continue to express dissatisfaction with local governments and demostrate low participation in governmental affairs. ^6^

In the 21st century, peace is redefined as an active process that goes beyond conflict avoidance, emphasizing the value of human dignity and mutual care for ourselves, others, and the planet. Educational systems play a crucial role in fostering this new understanding, by equipping individuals with the knowledge and skills needed to address climate crises sustainably, promote global citizenship through cooperation and dialogue, and advance gender equality by ensuring equal access to education. ^5^

Moreover, in the digital age, literacy is essential as it enables informed decisión-making, critical thinking, and ethical online behaviour. ^5^ Various authors have also recognized the concept of digital democracy, ^7^ a field still requiring significant research, especially with the rise of artificial intelligence, fake news, and hate speech on social media. ^8^

Cabezudo and Haavelsrud ^9^ established that to perform peace education processes, it is essential to start with a series of fundamental ideas. These include understanding the meaning of “peace,” the role of the community in its construction, and the expected impacts in the short, medium, and long term. Progress has been made in defining the concept of peace, moving away from the traditional idea of simply “the absence of violence and the presence of social justice, participation, and diversity” (p. 10). Peace is understood as a process of freedom that recognizes the learning capacity of each individual and the importance of the sociocultural context in which they find themselves. ^10^ Dialog plays a crucial role in building this culture of peace, as it is both an act of human recognition and an ontological and epistemological necessity to seek truth together with others. Thus, it is recognized that building a peaceful society is not solely the responsibility of the social sciences, but all disciplines must address the challenges and issues raised, maintaining a close link between education for peace and a culture based on respect for human rights, non-violence, and dialog, thus promoting peaceful relationships and continuous transformation. ^11^

Unde this context, an excellent approach is peace education based on a series of pillars that facilitate the construction of a culture of peace. These pillars are known as the dimensions of education for a culture of peace. ^12^ In this case, we focus on the dimension of democratic values, as mentioned by Cerdas-Agüero. ^13^ Democratic exercise must always be accompanied by values and good attitudes, with the main ones being predisposition, conviction, and willingness to act coherently and responsibly. Likewise, ethical reflection and the expression of values are crucial for democratic life, especially in societies facing crises of civility and lack of positive values, which can lead to demoralization and confusion.

Democratic values are a cornestone in the formation of citizens committed to building fair, equitable, and participatory societies. From a historical perspective, education for democracy has been considered a cornerstone in the construction of citizenship, understood as the ability of individuals to actively participate in the political and social processes that shape their environments. ^14^ In recent decades, debates on how educational institutions can promote democratic values have gained relevance in contexts marked by the rise of populism, ideological polarization, and the weakening of liberal democracies. ^15^^,^^16^

The literature on democratic values in education identifies multiple dimensions. On one hand, authors such as Westheimer and Kahne ^17^ emphasize the importance of a comprehensive approach that encompasses not only knowledge of rights and duties but also critical attitudes and skills. Within this framework, they propose three models of citizenship: responsible, participatory, and justice-oriented. Responsible citizenship involves adherence to basic norms and duties, while participatory citizenship encourages active engagement in the community. In the same way, justice-oriented citizenship aims to shape citizens capable of identifying and questioning unjust power structures, proposing alternatives for social transformation.

The conceptualization of citizenship has also evolved toward an approach that includes both political and social dimensions. Alexander ^18^ introduces the concept of “civil society” as a space where citizens interact not only with state structures but also with one another, sharing values and cultural meanings. This approach complements the classical view of citizenship as political participation by highlighting the importance of social cohesion and coexistence in diversity.

The school environment and curriculum play a crucial role in promoting democratic values. An open pedagogical climate, characterized by dialogue, inclusion, and respect for differences, has proven effective in developing civic competencies. ^19^^,^^20^ For instance, studies such as those by Leenders and Veugelers ^21^ show that classrooms encouraging open debate on controversial issues not only enhance students’ critical skills but also increase their willingness to engage in civic life.

Regarding the curriculum, specific citizenship projects and structured activities, such as simulations of democratic processes or community work, strengthen the connection between educational content and civic practice. ^22^^,^^23^ This approach is particularly relevant in multicultural contexts, where education in democratic values can serve as a powerful tool to promote integration and respect for diversity. ^24^

In the current context, education in democratic values faces significant challenges. The rise of populist and nationalist movements has contributed to the erosion of fundamental democratic values such as equality, justice, and respect for human rights. ^15^^,^^25^ Westheimer ^23^ argues that this situation calls for an educational response that not only promotes critical thinking but also inspires students to imagine and build more just societies.

Similarly, the Dublin Declaration by the Global Education Network Europe ^16^ reinforces this call, emphasizing that education should empower individuals to critically reflect on the world and act in favor of social justice and sustainability. This approach requires a paradigm shift in teaching methods, prioritizing participatory and dialogical practices that allow students to explore multiple perspectives and develop the skills needed to navigate social complexity.

In this way, science education plays a crucial role in fostering critical thinking, an essential skill for individuals to face contemporary challenges consciously and reflectively. It goes beyond the mere memorization of concepts, providing tools that enable individuals to question, analyze, and understand the world around them. ^26^ This understanding supports evidence-based decisión-making rather than assumptions. Moreover, science education is closely linked to civic education, as both empower individuals to comprehend societal issues more effectively and actively contribute to solving them. ^27^

In the current context of the triple planetary crisis—climate change, biodiversity loss, and pollution—science educators have the responsibility to cultivate a mindset in students and communities that integrates scientific knowledge with environmental respect and social accountability. ^28^^,^^29^

The benefits of this approach are evident. Individuals not only acquire practical skills such as problem-solving and teamwork but also evolve into more conscientious citizens, aware of the impact of their actions and motivated to help build a sustainable future. ^30^ By combining science education with a civic perspective, communities become more critical and engaged, capable of identifying local and global issues while proposing and leading effective solutions. Teaching science, therefore, transcends its academic purpose; it becomes a means of empowering individuals to take control of their circumstances and act as agents of change in a world that urgently requires innovative ideas and sustainable solutions.

In this way, natural science education offers a unique and innovate path to address these challenges, fostering critical thinking, collaborative problema-solving and evidence-based reasoning, skills essential for democratic participation. Nevertheless, it also presents major challenges when viewed through development and is disconnected from the broader context of social justice and sustainability, a call increasingly echoed from the perspective of global citizenship. ^31^

In the educational field, applied research in natural sciences has focused on analyzing scientific domains while neglecting the analysis of this field’s influence on social dynamics. Furthemore, the teaching of natural sciences tends to be fragmented, focused on disciplinary perspectives, and hindering a broader and interdisciplinary approach. ^32^ The aims of this article is to analyze the development of international literatura on promoting democratic values through natural science education. This will allow for planning future research, filling a gap in current literature, and answering the following question: How has the teaching of natural sciences facilitated the promotion of democratic values?

## Methods

In this study, the PRISMA (Preferred Reporting Items for Systematic Reviews and Meta-Analyses) methodology was adopted to ensure a rigorous and structured systematic review. ^33^ The specific objectives of the study were initially defined to guide the selection and analysis of relevant literature. Subsequently, the inclusion and exclusion criteria for the studies were clearly established to focus on the most pertinent and high-quality publications. ^33^ For data collection, exhaustive searches were conducted across Scopus and Web of Science databases using a detailed search strategy with Boolean equations, as shown in Table 1.

**Table 1.** Boolean equations.

No.	Boolean equation
1	(“Science“ OR ”Natural Science“ OR ”Environmental education”) AND “democratic values”
2	(“Natural Science“ OR ”Environmental education”) AND “democratic values”
3	(“Natural Science“ OR ”Environmental education”) AND “learning” AND “democratic values”
4	(“STEM“ OR ”STEAM”) AND (“curriculum“ OR ”curricula“ OR ”education“ OR ”learning”) AND “democratic values”
5	(“Science“ OR ” Natural science“ OR ”Environmental education”) AND (“curriculum“ OR ”curricula“ OR ”learning”) AND “democratic values”

**Figure 1.**
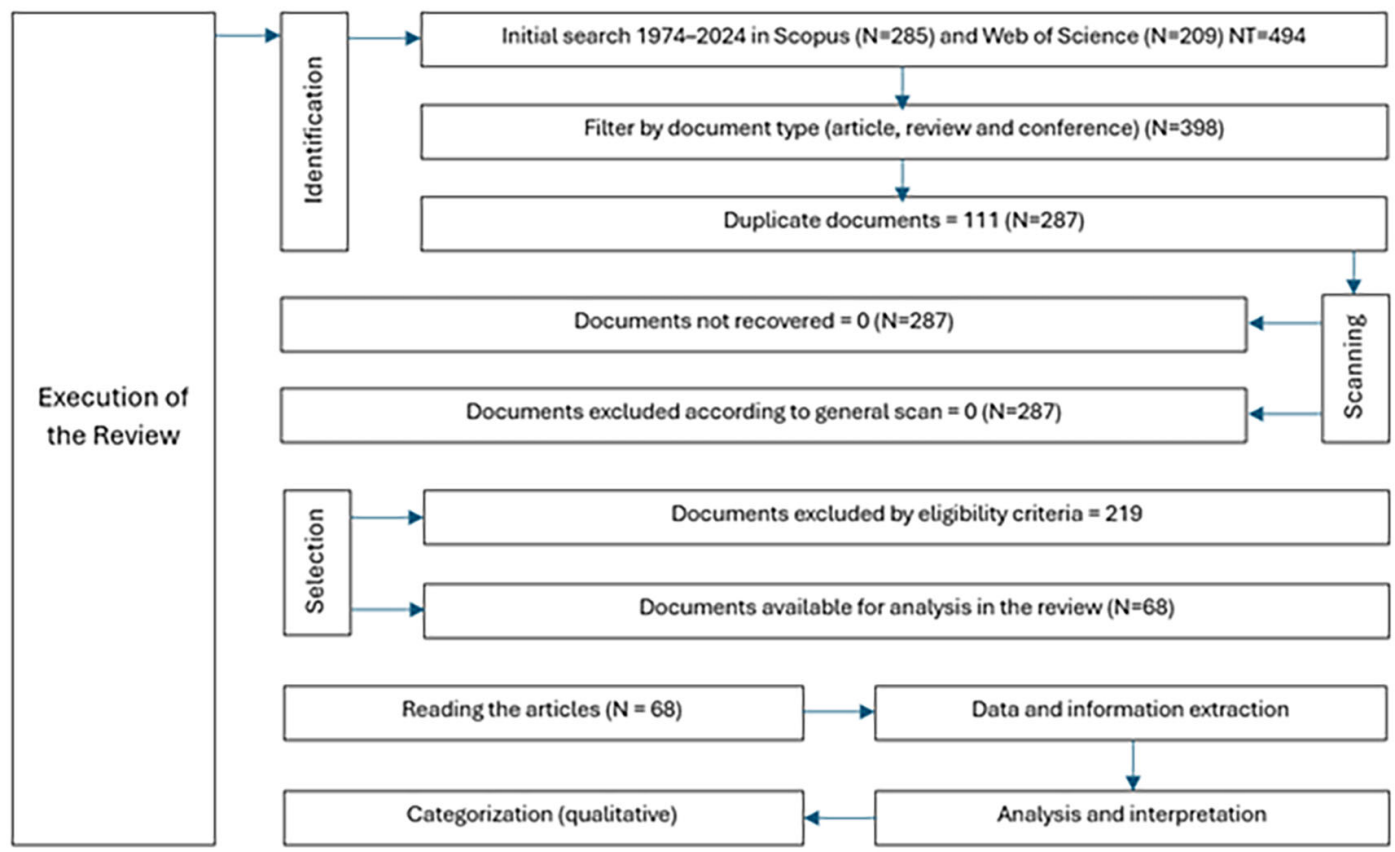
PRISMA flowchart for the literature review.

The study selection process was conducted using a two-stage approach, in which studies were initially filtered on the basis of titles and abstracts, followed by full-text evaluation for those that met the preliminary criteria: the relationship with democratic values and the relationship with education in natural sciences; the first filter focused on identifying studies that promote or evaluate the integration of democratic values such as equity, justice and citizen participation within the educational context, and the second filter addressed the specific connection with natural sciences education, seeking research that explores effective methodologies, content, and pedagogical practices in natural sciences teaching. This process is illustrated in a PRISMA flowchart ( Figure 1) that reflects the stages of selection and the reasons for study exclusion. Three reviewers performed a quick reading of the articles, focusing on summary, objective, methodology and results, they worked independently and then a second review was carried out analyzing the same criteria. Data extraction was performed independently by three reviewers to avoid bias, and any disagreements were resolved through discussion.

The extracted data were synthesized using tools such as ScientoPy ^®^ version 2.1.3 and VOSviewer ^®^ version 1.6.20 for preprocessing, subsequently facilitating the identification of clusters and interactions among the contents and authors. Finally, a qualitative analysis was conducted where six categories were defined, with each article being classified into a category, thus providing a comprehensive and systematic assessment of the existing literature, yielding well-founded conclusions.version 1.6.20 for preprocessing, subsequently facilitating the identification of clusters and interactions among the contents and authors. Finally, a qualitative analysis was conducted where six categories were defined, with each article being classified into a category, thus providing a comprehensive and systematic assessment of the existing literature, yielding well-founded conclusions.

To ensure the methodological rigor of this study under the PRISMA guidelines, the methods used to assess the risk of bias in the included studies were detailed thoroughly. Multiple independent reviewers were assigned to each study to ensure objectivity, and in cases of discrepancies, a third reviewer was consulted. Automated tools were utilized when necessary, particularly in the analysis and construction of clusters.

Regarding the synthesis methods, data extraction processes were employed, identifying primary outcomes, categorizing results, and deciding the eligibility of studies for each synthesis. This included tabulating the characteristics of each article and comparing them with the planned categories.

In terms of data preparation for presentation or synthesis, all articles were downloaded and systematically organized in MS Excel, where the analysis and categorization processes were conducted. For tabulating and visualizing results, the previously described tools were used to present the results of individual studies and syntheses. Sensitivity analyses were conducted, and possible causes of heterogeneity among the results were explored. For assessing the risk of bias due to missing results, a comprehensive reading of the findings with artificial intelligence was performed to confirm the identified ideas.

The data from the literature review process, integration of democratic values in natural sciences education, the PRISMA checkboard and the process flow diagram have been published by Ref. 34 and are located in the availability section.

## Results

According to the methodology outlined above, the following section presents an overview of the results obtained from the preprocessing, the networks constructed with the selected articles, and the findings from each category.

### Current trends identified

**Figure 2.**
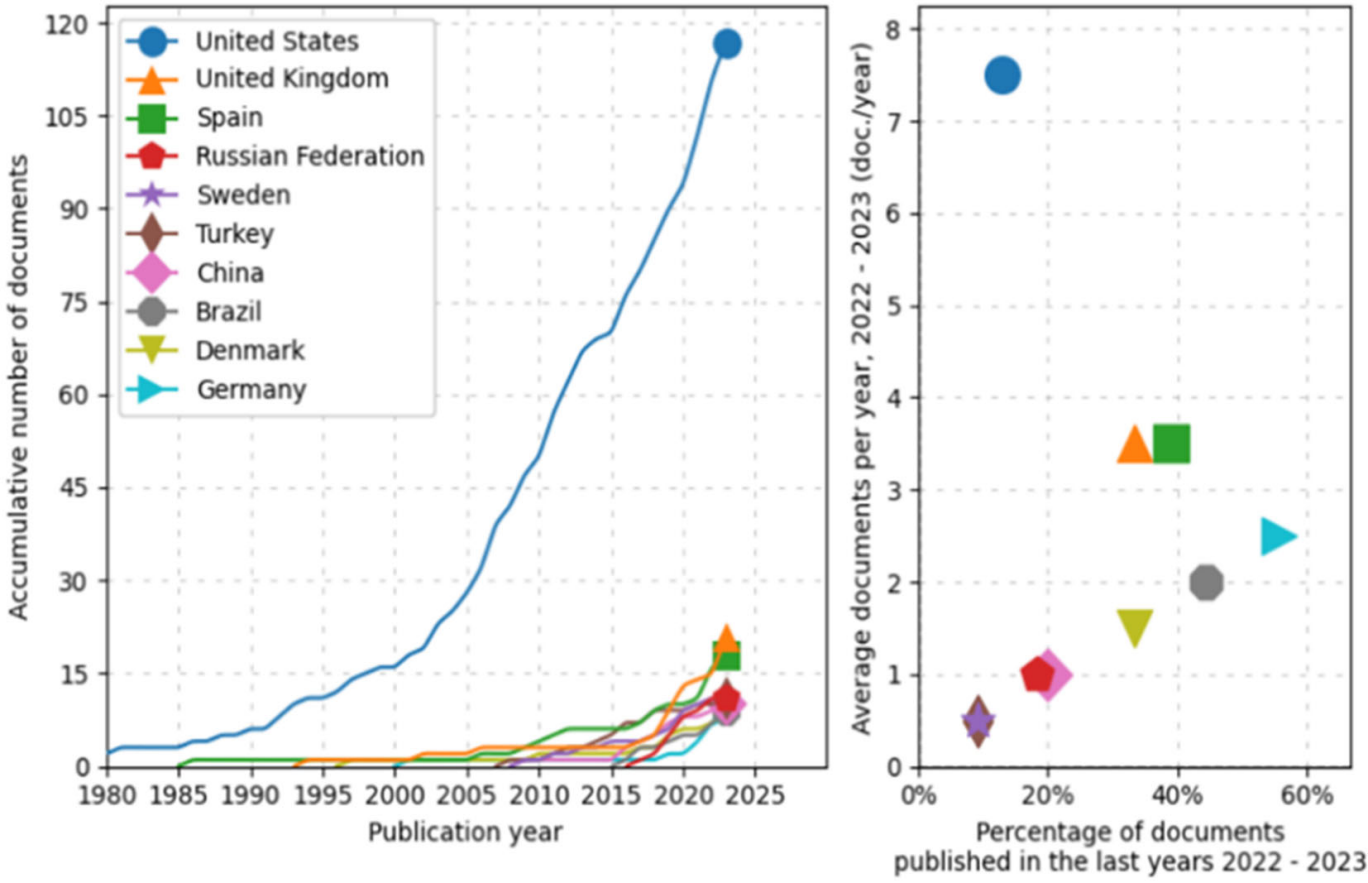
Scientific production. Source: the authors, made in ScientoPy.

First, during preprocessing, the evolution and distribution of scientific production from various countries over time is identified, as shown in Figure 2. The line graph on the left shows an exponential growth in scientific document production from the United States, particularly noticeable since 2000. In comparison, other countries such as the United Kingdom, Spain, the Russian Federation, Sweden, Turkey, China, Brazil, Denmark, and Germany also show an increase in the number of published documents, albeit more moderately. Notably, nations like China and Brazil have significantly intensified their document production in the last two decades.

The scatter plot on the right illustrates the recent activity (2022-2023) of these countries in terms of scientific publications. Although some countries show a lower percentage of recently published documents, the average number of documents published per year during this period is relatively high for some, a recent intensification in their research efforts. This analysis not only allows appreciation of the historical trajectory of scientific production from the selected countries but also helps identify current trends in their contributions to the global scientific corpus.

**Figure 3.**
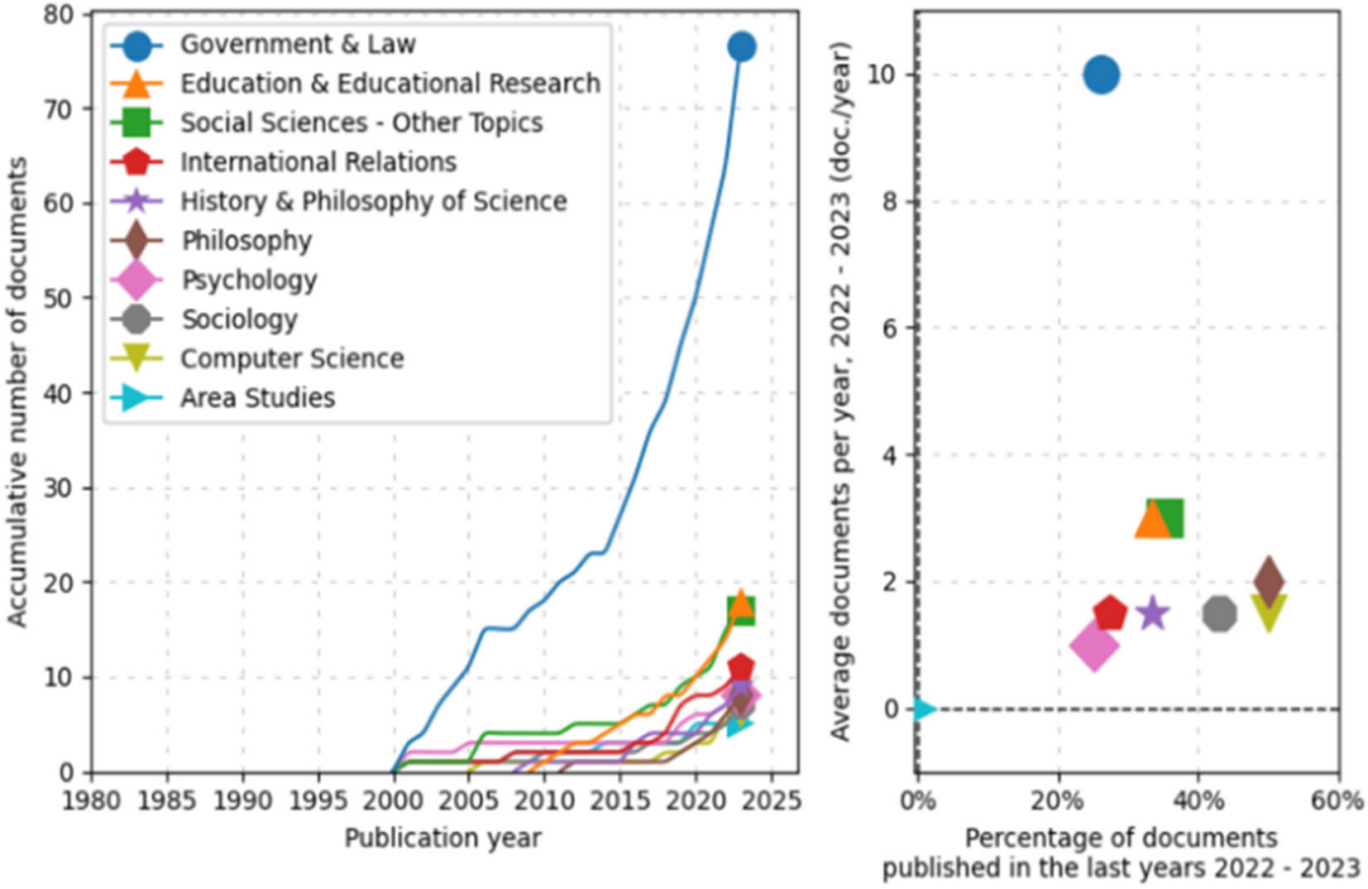
Trends by topic. Source: the authors, made in ScientoPy.

Subsequently, an analysis of trends by topic in documentary production from 1980 to 2025 was conducted, as shown in Figure 3. In the line graph, the field of “Government & Law” has experienced significant exponential growth, particularly starting in 2005 and continuing with a marked increase until 2025. Other fields such as “Education & Educational Research,” “Social Sciences-Other Topics,” and “International Relations” also show a steady increase, albeit less pronounced.

Regarding the scatter plot, which assesses the recent activity of these fields in 2022-2023, it can be seen that “Government & Law” not only dominates in terms of cumulative growth but also in recent annual average production, indicating a high ongoing activity in this field. Other fields, while having fewer cumulative documents, show a respectable average of recently published documents per year, a renewed or emerging interest in these topics.

**Figure 4.**
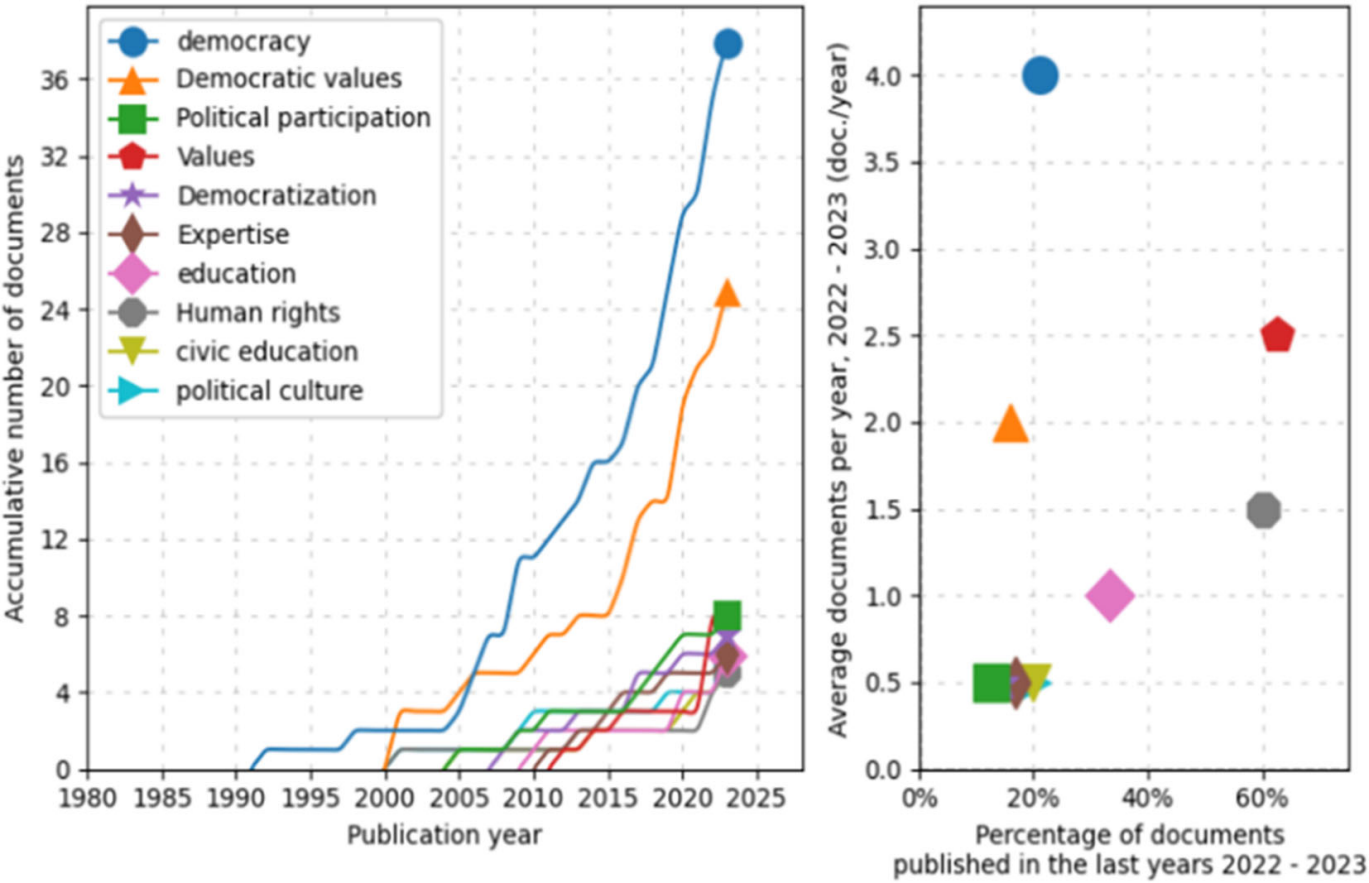
Trends by key words. Source: the authors, made in ScientoPy.

Similarly, a trend analysis using keywords was conducted, as shown in Figure 4. First, in the line graph on the left, the field of “democracy” shows a prominent exponential growth, especially after 2005, peaking in 2025. Other topics such as “Democratic values”, “Political participation”, and “Democratization” also exhibit a notable increase over time, although on a more moderate scale. Fields like “education” and “Human rights” demonstrate steady but less dramatic growth.

In the scatter plot on the right, which assesses recent publication activity (2022-2023) in these topics, it is evident that “democracy” not only leads in terms of cumulative growth but also maintains a high average of documents per year in the recent period, indicating a sustained and robust interest in studies on democracy. Topics such as “Democratic values” and “Political participation” show a significant percentage of recent documents, reflecting renewed focus on these themes in contemporary literature.

**Figure 5.**
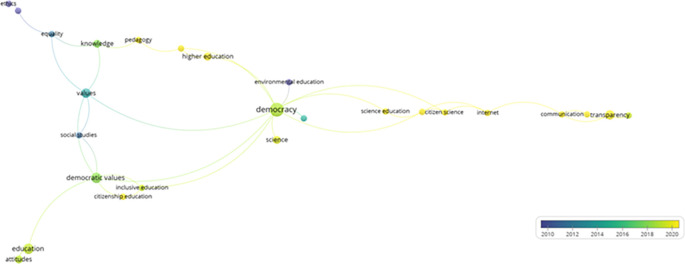
Key words network. Source: the authors, made in VosViewer.

Subsequently, from the selected articles that help to address the research question, a network of keywords was constructed in VOSviewer ( Figure 5), demonstrating how various themes related to education, democracy, and science are interconnected across seven main clusters.

The first cluster emphasizes citizenship education and encompasses aspects such as democratic values and inclusive education, highlighting the importance of integrating democratic principles into educational training to foster active social participation. The second cluster explores the relationship between science and citizenship, underscoring the role of the Internet in scientific dissemination and how it affects communication and transparency in the public sphere.

The third cluster links civics education with higher education and knowledge, proposing a synergy between civic training and advanced pedagogy. Meanwhile, the fourth cluster focuses on communication via social media, examining topics such as epistemic trust and transparency, and how these factors influence public perception and trust toward institutions. The fifth cluster connects democracy with environmental education and evolutionary biology, illustrating how educational approaches in science can complement and enhance understanding and participation in democratic practices, especially in the context of sustainability.

The sixth cluster addresses ethics, equality, and learning, underlining the relevance of these values in education and their impact on social and cultural development. Finally, the seventh cluster focuses on attitudes and education, exploring how predispositions toward learning and teaching affect educational processes.

This network provides a comprehensive view of how the interaction between these themes can generate new perspectives and opportunities for integrating the concepts of democracy, science, and education. It also highlights the importance of interdisciplinarity in the study of education and democracy, showing how different fields can collaborate to promote a more informed and participative society.

**Figure 6.**
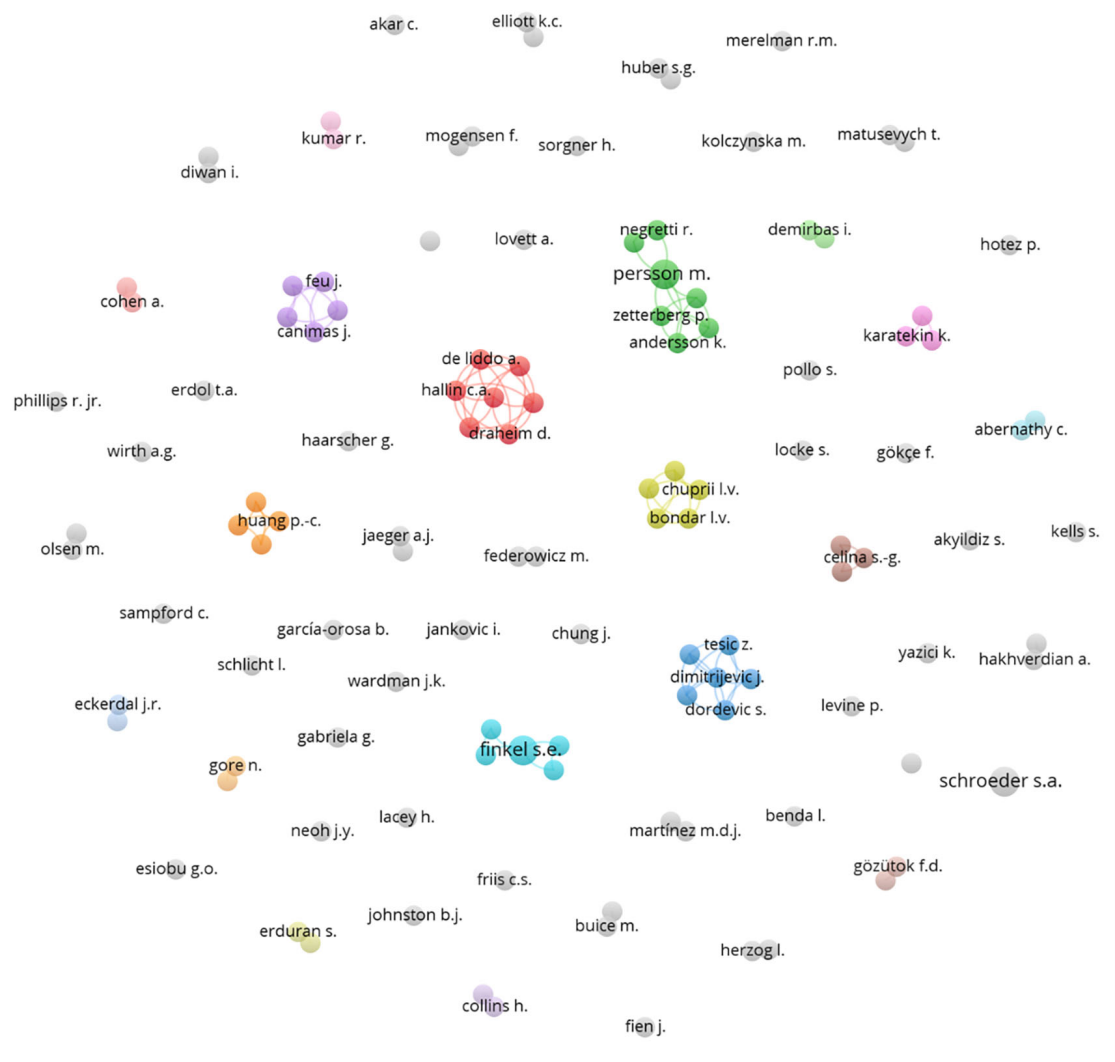
Authors network. Source: the authors, made in VosViewer.

A network of collaborations among various authors was subsequently constructed ( Figure 6), represented by several colored nodes. These nodes indicate groups of authors who frequently co-publish or are thematically related. Each node in the diagram represents an author, and the lines connecting the nodes indicate collaborations between them. The different colors assigned to the nodes may represent different fields of study, institutions, or simply co-authorship groups that have collaborated closely on several research projects.

The network shows low collaboration among various researchers, indicating a limited shared interest in integrating democratic values into science education. This organization suggests a multidisciplinary approach, where different aspects of science and democracy are addressed from various angles without effective integration.

The red cluster shows a group of authors who collaborate closely with each other, suggesting a cohesive approach and possibly a joint project or a series of publications related to specific topics within the study of democracy and natural sciences. Other clusters that demonstrate collaboration are the green, light blue, and orange clusters.

Each study was independently reviewed by two reviewers, and in cases of discrepancy, a third reviewer intervened to reach a consensus. The risk of bias assessment focused on aspects such as category selection, result extraction, outcome assessor blinding, and the handling of incomplete data. For the synthesis of the studies, key characteristics such as study design, sample size, educational context, and main outcomes were analyzed. The included studies varied in terms of methodological approach. Most studies showed a low risk of bias in critical areas.

Possible causes of heterogeneity among study results were investigated through collaborator network analysis and cluster analysis with keywords. Differences in contexts and methodologies used were the main sources of heterogeneity. Additionally, studies that used qualitative versus quantitative approaches showed heterogeneous results in terms of effectiveness and perception of democratic values.

### Development of teaching strategies, methodologies, and techniques

In the first instance, this category aims to classify articles that discuss and/or propose innovative approaches to teaching natural sciences in conjunction with democratic values, including innovative pedagogical methods and the integration of technologies in the classroom through interdisciplinary approaches. Initially, educational theory and philosophy were considered as the academic foundation for the relationship between education and politics, portraying the teacher and their methodologies as symbols of empowerment and social change, allowing for a series of criticisms and innovations in the educational systems of countries around the world, prioritizing students and their role in society. ^35^ The development of these methodologies has progressed as education was initially conceived as an alternative for social development (which is not wrong, but nowadays it is not limited solely to this field), incorporating democratic values into the school curriculum ^36^; Likewise, these types of models have been reconsidered over time, demonstrating how education provides the necessary tools to develop a democratic reflection exercise, thus promoting critical and thoughtful citizenship by integrating all disciplines, in this case, the natural sciences. However, Abernathy and Forestal ^37^ suggest that the integration of education with democratic values should be carried out through extracurricular activities, as this is the context in which its impact on political knowledge, civic skills, and values can be assessed, offering a useful model for implementing effective and accessible civic learning programs. Erduran and Kaya ^38^ proposed a series of questions for science teachers to promote scientific skills to achieve the goal of a critical and integral society regarding democratic values and how it can be achieved through various pedagogical models proposed in the research.

It is also important to recognize what Plaza De La Hoz ^39^ proposed, whose investigation delves into the evolving dynamic between educational authority and democratic values considering the integration of Information and Communication Technologies (ICT). Its dual objective is to analyze recent scholarly works concerning the impact of ICT on pedagogical authority and to corroborate these insights through empirical observation. Contrary to prevalent critical perspectives, the investigation posits that ICT serves to bolster teacher authority, albeit in a transformed manner, transitioning from a predominantly authoritative stance to one that is more demonstrative. Thus, corroborating the way in which not only education but also its approaches, strategies, and didactics have been advancing alongside political and social development.

### Transformative experiences in the promotion of democratic values in natural sciences

Contrary to what has been exposed, but to great surprise, methodologies with a positive impact have not stood out according to this study, since only one investigation belonging to Esiobu ^40^ has been found. This study investigates the impact of cooperative learning on promoting peace, equality, and equity in science classrooms, which are essential for sustainable development within a democratic framework. Through a gender equity and peace questionnaire, we found that cooperative learning effectively fosters equity and peace among genders in the biology classroom. This establishes a strong relationship between the Sustainable Development Goals and the development of scientific thinking in the classroom, thus promoting democratic values within the institution.

On the other hand, Persson and others ^41^ conducted an experimental study on deliberative education, comparing its effects with traditional teacher-centered education by evaluating four forms of civic competence: political knowledge, political interest, democratic values, and political discussion. The results showed that deliberative education does not significantly enhance civic competence. However, it is important to explore effective pedagogical approaches in both civic education and science education that not only convey knowledge but also foster interest, critical discussion, and ethical values. ^41^

### Presence of democratic values in scientific formal education programs

The integration of science into educational programs has become pivotal for developing scientific skills within communities, particularly in areas concerning the environment and community health. Through cienciometric tracking, a wide array of documents emphasizing the significance of promoting environmental education for ecosystem conservation and fostering democratic values for sustainable development have been identified. ^42^ However, this challenge is not confined to classrooms; it also poses a dilemma for policymakers, as environmental governance is essential for devising strategies that safeguard the environment and biodiversity through effective governance. ^43^

Presently, environmental education is on the rise, with natural sciences focusing on sustainable applications and explanations of environmental phenomena. This interdisciplinary field permeates all areas of knowledge because of its versatility and is closely linked to democracy and its values. Terms such as democracy and environmental justice are addressed, empowering educators, students, and policymakers to reconsider their impact on preserving the world. ^44^ Environmental education is incomplete without action, as highlighted by Mogensen and Schnack, ^45^ who underscore the crucial role of action in sustainable development processes in society. Since 1980, this component has been integrated into Denmark’s educational programs, resulting in significant shifts in population dynamics and bolstering democratic values regarding the environment across all knowledge domains.

Despite science being fundamental for societal development, uncertainties persist regarding sustainable development, biodiversity conservation, and the contribution of democratic values to achieving the Sustainable Development Goals. ^46^ The emergence of new technologies offers opportunities for scientific and social innovation, prompting educational and governmental institutions to develop action plans to consolidate scientific knowledge within communities. It is imperative to not overlook the importance of democratic values in ensuring global sustainable development. ^47^^,^^48^

### Democracy: a journey

The importance of integrating democratic values into various fields of knowledge has been emphasized; however, it is crucial to specify which types of democratic values should be promoted through different research. Initially, these values must be enhanced through active participation; nevertheless, highlighted challenges in student motivation, fewer female candidates and representatives, and gender biases favoring male candidates have been identified. Female students faced obstacles and lacked democratic campaign methods; however, they challenged gender norms by supporting female candidates. ^49^

Similarly, new technologies pose a fresh challenge to democratic values. The emergence of digital disruptions, particularly facilitated by platforms like social media, presents a notable obstacle to democratic principles and collective wisdom. Concerns such as misinformation, political biases, and increasing distrust in institutions have the potential to undermine the efficacy of collaborative endeavors. Safeguarding the pivotal role of collective participation in confronting contemporary challenges requires immediate action to address issues like inclusivity, openness, and group dynamics. ^50^ Recent surges in antivaccine activism and other antiscience trends have converged with rising antisemitism. A clear example of this phenomenon arose during the COVID-19 pandemic, as far-right elements often employed Nazi imagery to criticize vaccinations and even blamed the Jewish people for COVID-19 and vaccine profiteering. These parallels with historical persecution underscore the urgent need for action. ^51^

Moreover, democratic values are linked to science in society. Initially, the focus was on reflecting on the relevance of scientific disciplines in democracy, and vice versa, integrating the foundations of both natural and social sciences, reaching scientific and democratic philosophy, and presenting points of disagreement or agreement. ^52^ In essence, both fields of knowledge are interdependent, considering that natural sciences provide explanations for phenomena that facilitate or challenge human development; likewise, social sciences provide knowledge regarding human behavior and society, relating aspects such as justice, equity, and recognition of various ethnicities and populations.

These sciences are at the forefront of exercising democratic values concerning the environment, gender, and sustainable development. ^53^ Thus, in democratic societies, the threat of populism persists, but science can help mitigate this risk. By upholding shared values like honesty and universalism, science serves as moral leadership, preventing the rise of populism. Additionally, science contributes to a system of checks and balances that limit executive power. However, an excess or deficiency of science can lead to democratic failures, highlighting the need to reaffirm democratic values.

Meanwhile, a study on tenured academics’ experiences in science communication reveals their commitment to sharing knowledge beyond academia, despite the challenges it poses to their academic work. They engage in various writing practices driven by a desire to educate and democratize scientific knowledge. Further research and training programs are required to enhance science communication skills among future scientists. ^54^^–^^56^ In this regard, Manias and others ^57^ emphasize the necessity of developing reliable, transparent, and accountable systems to reinforce values ​​supported by Artificial Intelligence (AI). Similarly, in education, it is essential to promote an understanding of how technology can and should be ethically and responsibly designed and used. This fosters critical engagement in debates about technology, ethics, and society and contributes to the creation of technologies that benefit society inclusively and equitably.

### Civic education

Civic education seeks to promote critical awareness of social, political, and environmental issues, fostering respect for diversity and a commitment to the common good. As noted by Matusevych and Shevchuk, ^58^ civic education is a fundamental tool for building trust across various domains, with the aim of developing social responsibility grounded in ethical principles and the pursuit of the common good. This approach not only aims to inform students about their rights and duties as citizens but also motivates them to actively participate in society and contribute to its improvement.

Similarly, education for citizenship focuses on preparing citizens to navigate the dynamism of modern societies. According to Neoh, ^59^ it is crucial that curricula are designed to reflect the objectives and developmental needs of contemporary society. This necessitates a constant adaptation of educational content to ensure that students are well equipped to face contemporary challenges, such as globalization, digitalization, and environmental changes.

In addition, research underscores the importance of fostering civic responsibility within higher education institutions. As highlighted by Swidler’s framework on civic responsibility, educational institutions can promote democratic values by emphasizing five key dimensions: knowledge of and support for democratic systems, commitment to community welfare, application of knowledge for societal benefit, appreciation of diversity, and personal accountability. These principles provide a more cohesive and impactful approach to civic education. ^60^

However, Feu et al., ^61^ underscored the importance of addressing key concepts in a unified manner within the educational sphere. According to this study, for the teaching-learning process to be effective, it is essential that all educational actors (teachers, students, administrators) share a common and cohesive understanding of the principles and objectives of civic education. This ensures that the values and knowledge imparted are coherent, facilitating their internalization and practical application by students.

### Teacher training

In this category, strategies for initial and ongoing teacher training are analyzed to prepare educators for classroom challenges by integrating democratic values such as equality, inclusion, and mutual respect into their pedagogical practice. Johnston ^62^ suggests that leadership, the pursuit of democratic values, and the structural and cultural dimensions of school organization are fundamental for continuous societal improvement. It is crucial to define the roles of formal school leaders, teachers, and other school staff to create an educational environment that supports democratic objectives.

Additionally, Yazici ^63^ presented a study on the democratic values of prospective teachers using the Democratic Teacher Values Scale. The study indicates that future teachers exhibit high levels of democratic values in terms of solidarity, the right to education, and freedom. This study underscores the need to incorporate these values into teacher training programs so that teachers can effectively promote a learning environment that reflects these principles. It concludes that factors such as the university attended and the father’s educational background significantly impact these values, highlighting the importance of a comprehensive and contextualized approach to teacher training.

Finally, Gökçe ^64^ and Dogru and Demirbas ^65^ explored teachers’ perceptions and expectations regarding the parameters of rights, freedoms, and responsibilities within democracy. Gökçe revealed a significant discrepancy between the observations and expectations of prospective teachers concerning these values, indicating an urgent need to align educational practices with democratic ideals. On the other hand, Dogru and Demirbas found a positive and moderate relationship between teachers’ perceptions of multicultural competence and their democratic values, suggesting that strengthening these values can enhance teachers’ ability to manage classroom diversity. Both studies emphasize the importance of teacher training that not only imparts knowledge but also fosters a culture of justice, respect for differences, and equity.

Some findings emphasize the critical role of teacher training in fostering trust and democratic responsibility within educational systems. The study by Cohen and Gilead ^66^ highlights the importance of preparing educators to effectively manage diverse classrooms while simultaneously upholding democratic and ethical values. Similarly, Chung and Akyildiz ^67^ propose the integration of multicultural perspectives into teacher training programs, aiming to better equip educators to address the challenges posed by increasingly diverse societies.

## Discussion

First, there is a clear convergence on the notion that education should transcend the mere transmission of technical knowledge, incorporating democratic values essential for the development of a more just and participatory society. The integration of ICT, civic education, and service-learning are prominent strategies that promote equity, participation, and the moral and intellectual development of students. Scientific argumentation and deliberative democracy are presented as complementary methods that enrich teaching and foster critical and participatory understanding among students.

Friis ^36^ and Plaza de la Hoz ^39^ highlight how ICT can enhance equity and participation in education, demonstratively reinforcing pedagogical authority. Abernathy and Forestal, ^37^ along with Iskhakova et al., ^68^ emphasize the need for civic and political education to cultivate informed and engaged citizens by employing both extracurricular events and innovative approaches to political education. Wirth ^69^ and Erduran and Kaya ^38^ explored the intersection between science and democracy, proposing that scientific education should include practices of argumentation and democratic deliberation to develop critical and participatory skills. Finally, Óscar, Celina, and Jesús ^70^ advocated service-learning as a methodology that combines academic learning with community service, fostering social responsibility and civic engagement.

Feu et al. ^71^ addressed the need for a precise and multifaceted understanding of democracy within the educational sphere, highlighting four dimensions: governance, habitance, alterity, and ethos. This framework can be applied to science education to promote an inclusive and participatory school culture, thereby fostering ethical responsibility and respect for diverse perspectives.

Thornton and Jaeger ^60^ complement this vision by emphasizing civic responsibility within higher education. They argue that promoting scientific knowledge and skills for societal benefit and appreciation for diversity are pertinent to science education, which can contribute to forming students aware of their societal roles.

Conversely, Purevdagva et al. ^72^ underscored the importance of fact checking and critical thinking in scientific education. Educating students about the significance of critically evaluating and understanding the world is essential for fostering informed and engaged citizenship.

Herzog and Lepenies ^73^ mention that citizen science can promote deliberative democracy and address epistemic injustice. Integrating active student participation in dialog about the direction and impact of scientific research encourages scientifically informed and engaged citizens.

Esiobu ^40^ demonstrated how cooperative learning in science classroom can promote gender equity and inclusion. By showing that this methodology can overcome gender barriers and promote equality, cooperative learning is essential for democratic education.

Additionally, Federowicz and Terepyshchyi ^74^ support this vision by highlighting how integrating democratic values into the Polish educational system can facilitate cultural understanding, civic engagement, and social inclusion. As Thornton and Jaeger ^60^ mention, civic responsibility within higher education is an approach applicable to natural sciences that promotes the use of scientific knowledge for societal benefit and fosters an appreciation for diversity.

However, Sorgner ^75^ discusses the relationship between scientific authority and democratic participation in decision making. He addresses the balance of expert knowledge with the inclusion of diverse perspectives and democratic values in scientific education to prepare students for engaging in critical dialogs about science and society.

Manias et al. ^57^ discussed the need for transparency and accountability in the use of AI in governance. They emphasize the importance of educating natural science students about ethical and democratic principles concerning technology, promoting a critical understanding of how technology can be ethically and responsibly designed and used.

As Schroeder ^76^ mentions, science should appeal to democratic values, meaning the values of the public or their representatives, to maintain or even strengthen public trust in scientific research. He emphasizes the importance of integrating democratic values into scientific education.

In brief, it is observed that a significant portion of the reviewed studies focuses on developing innovative approaches to teaching natural sciences while simultaneously promoting democratic values. This category encompasses pedagogical methodologies that go beyond merely imparting scientific knowledge, emphasizing the promotion of values such as equality, inclusion, and active participation within a democratic framework.

The initial focus of many studies is rooted in educational and philosophical theory as an academic foundation for the relationship between education and politics. This approach presents teachers and their methodologies as symbols of empowerment and social change, representing a significant evolution in the educator’s role. No longer limited to transmitting information, educators are now tasked with fostering critical and reflective citizens. This transformation has profound implications for educational systems, which prioritize students not only as recipients of knowledge but also as active agents in society.

In this regard, Erduran and Kaya ^38^ proposed a series of questions aimed at science teachers to promote scientific skills that enable the development of critical and holistic citizenship, aligning with democratic values. This initiative highlights the importance of pedagogical models being oriented not only toward the acquisition of technical knowledge but also toward the formation of committed citizens aware of the role of science in society. Additionally, an interesting perspective is provided on the evolution of educational authority concerning democratic values, considering the integration of Information and Communication Technologies (ICT). ^39^ This has profound implications for how teachers exercise their role in the classroom, suggesting that ICT can enhance the teacher’s authority by enabling greater access to information and tools that facilitate learning.

Contrary to what was previously stated, but surprisingly, most methodologies with a positive impact were not significantly highlighted in the analyzed studies. Esiobu’s study ^40^ examines the impact of cooperative learning on promoting peace, equality, and equity within the science classroom. The results indicate that this type of learning effectively fosters equity and peace between genders, particularly in the context of biology classes. This suggests that cooperative learning can be a powerful tool for achieving Sustainable Development Goals (SDGs), ^24^ especially those related to gender equality and quality education.

Consistent with this, the integration of sciences into educational programs has become essential for developing scientific skills within communities, particularly regarding environmental and community health issues. In this context, environmental education emerges as a crucial component for promoting the democratic values necessary for sustainable development. ^25^^–^^27^

Thus, the integration of democratic values into education has significant implications for educational policies and pedagogical practices. To develop a more equitable and democratic society, it is essential to implement policies that promote equity and accessibility in education using innovative technologies and methodologies. Furthermore, fostering civic and political education from an early age is crucial for developing informed and engaged citizens.

In this way, the practices of democratic deliberation and argumentation in scientific education can enrich students’ understanding of the interconnection between science and democracy, preparing them to actively participate in scientific and political debates. Similarly, service-learning, as a pedagogical methodology, offers a practical approach to integrating academic learning with community service, promoting democratic values, and developing personal and professional competencies in students.

Initial findings and theories from various authors indicate that global citizenship education (GCE) is on the rise, aiming to prepare individuals to actively participate in an interconnected world. This educational approach faces the challenge of integrating natural sciences into its curriculum, especially in a context marked by the triple crisis—social, economic, and environmental—as well as the emergence of artificial intelligence and globalization. These factors are essential for the development of civic and scientific knowledge within the community, as they allow for addressing contemporary issues from a critical and holistic perspective. ^77^^,^^78^

The incorporation of natural sciences into GCE not only seeks to break down barriers between social and natural sciences but also to foster a deeper understanding of the interrelations between these fields. Scientific education should be contextualized within current social and environmental challenges, enabling students to understand how their actions impact the environment. According to UNESCO, ^79^ this approach is fundamental for forming committed and aware citizens capable of addressing complex problems through the development of critical and collaborative skills.

Finally, research in this area opens new possibilities for citizens to develop a global scientific mindset that integrates democratic values and sustainability principles. This involves not only acquiring technical knowledge but also fostering critical awareness of issues such as sustainable development and social justice. GCE should empower individuals to act as agents of change in their communities, promoting active participation in decision-making processes that affect their environment. ^80^^,^^81^

## Conclusions

The findings highlight that promoting democratic values through natural sciences education is an under-researched yet essential area. This process involves fostering critical thinking, gender equity, ethical responsibility, and civic participation, enhancing both scientific education and students’ roles as informed and active citizens in democratic societies. The study underscores the need to integrate democratic values into curricula and educational policies, emphasizing the role of information and communication technologies (ICT) in fostering equity and participation. Civic education is also crucial for enabling students to critically evaluate information and engage in scientific and political debates. The integration of approaches like scientific reasoning, democratic discourse, and service learning illustrates that science education must transcend technical aspects, fostering equity, inclusiveness, and active engagement. Nonetheless, the analysis indicates that these approaches remain inadequately addressed in academic literature, hence presenting an opportunity for future research.

Incorporating democratic deliberation and active student participation in scientific research strengthens the connection between science and social values, preparing students to navigate the interplay between scientific decision-making and democratic principles. Early and deliberate efforts to link science with civic and political education can enrich students’ understanding of democracy and empower them to contribute to more equitable and participatory societies.

### Ethics and consent

Ethical approval and consent were not required.

## Data Availability

No data associated with this article. S. Monsalve-Silva, J. G., Otalvaro-García, L. S, Cajica Velandia, & A. D., Vargas Sánchez. Extended data Integration of democratic values in natural sciences education [Data set]. Zenodo. 2024. doi: https://doi.org/10.5281/zenodo.13229007. ^34^ The project contains the following extended data:
1.

Flow_diagram_Integration of democratic values in natural sciences education.png
2.
Literature Data Integration of Democratic Values.xlsx
3.

PRISMA_Checklist_Literature Data Integration of Democratic Values.docx Creative Commons Zero v1.0 Universal
