# Supplementary material for: Integration of Democratic Values ​​in Natural Sciences Education: a Review of the Literature of the Last 50 Years
Source: F1000Res. 2025 Sep 12;13:940. Originally published 2024 Aug 20. [Version 4] doi: 10.12688/f1000research.154069.4 (PMC12413607; doi:10.12688/f1000research.154069.4)
Supplement: Peer review history

# Reviewer report: Reviewer response for version 4

**DOI:** 10.5256/f1000research.187850.r413755

**Published:** 2025-09-23

**Authors:** John Mart Elesio

**Affiliations:** 1 Holy Cross of Davao College, Davao, Philippines

The research article is good for publication.

Are the rationale for, and objectives of, the Systematic Review clearly stated?

Yes

Is the statistical analysis and its interpretation appropriate?

Not applicable

If this is a Living Systematic Review, is the ‘living’ method appropriate and is the search schedule clearly defined and justified? (‘Living Systematic Review’ or a variation of this term should be included in the title.)

Yes

Are sufficient details of the methods and analysis provided to allow replication by others?

Yes

Are the conclusions drawn adequately supported by the results presented in the review?

Partly

Reviewer Expertise:

Science Education

I confirm that I have read this submission and believe that I have an appropriate level of expertise to confirm that it is of an acceptable scientific standard.

## References

[1] : Integration of Democratic Values ​​in Natural Sciences Education: a Review of the Literature of the Last 50 Years. *F1000Research* .2025;13: 10.12688/f1000research.154069.4 10.12688/f1000research.154069.4 PMC1241360740919446

# Reviewer report: Reviewer response for version 3

**DOI:** 10.5256/f1000research.179956.r386518

**Published:** 2025-09-05

**Authors:** John Mart Elesio

**Affiliations:** 1 Holy Cross of Davao College, Davao, Philippines

This research aims to identify and analyze strategies, methodologies, and transformative experiences that contribute to the promotion of democratic values.

 Please align your conclusions to the objective of the study. What are the strategies and methodologies develop?  What transformative experiences that the study find out? Please elaborate how the research be useful to the reader.

Are the rationale for, and objectives of, the Systematic Review clearly stated?

Yes

Is the statistical analysis and its interpretation appropriate?

Not applicable

If this is a Living Systematic Review, is the ‘living’ method appropriate and is the search schedule clearly defined and justified? (‘Living Systematic Review’ or a variation of this term should be included in the title.)

Yes

Are sufficient details of the methods and analysis provided to allow replication by others?

Yes

Are the conclusions drawn adequately supported by the results presented in the review?

Partly

Reviewer Expertise:

Science Education

I confirm that I have read this submission and believe that I have an appropriate level of expertise to confirm that it is of an acceptable scientific standard, however I have significant reservations, as outlined above.

# Reviewer report: Reviewer response for version 3

**DOI:** 10.5256/f1000research.179956.r376393

**Published:** 2025-05-03

**Authors:** Anissa Lestari Kadiyono

**Affiliations:** 1 Universitas Padjadjaran, Bandung, West Java, Indonesia

This article is included in the Research on Research, Policy & Culture gateway. Therefore, it is necessary to discuss from the perspective of cultural fields and policies that can be produced from this research.

Are the rationale for, and objectives of, the Systematic Review clearly stated?

Partly

Is the statistical analysis and its interpretation appropriate?

Yes

If this is a Living Systematic Review, is the ‘living’ method appropriate and is the search schedule clearly defined and justified? (‘Living Systematic Review’ or a variation of this term should be included in the title.)

Yes

Are sufficient details of the methods and analysis provided to allow replication by others?

Yes

Are the conclusions drawn adequately supported by the results presented in the review?

Partly

Reviewer Expertise:

Psychology; Industrial & Organizational Psychology; Gender; Human Capital; Organizational Behavior

I confirm that I have read this submission and believe that I have an appropriate level of expertise to confirm that it is of an acceptable scientific standard, however I have significant reservations, as outlined above.

# Reviewer report: Reviewer response for version 3

**DOI:** 10.5256/f1000research.179956.r376394

**Published:** 2025-04-30

**Authors:** Genç Osman ilhan

**Affiliations:** 1 Yildiz Teknik Universitesi, Istanbul, İstanbul, Turkey

I have checked the revisions of the author and I would like to inform you that the revisions are made appropriately. The article can be approved now, based on the criteria and journal's principles.

 I hope this information is helpful to the author. I would like to congratulate the author for fulfilling every revision and providing a richer literature both in the introduction and discussion parts.

Are the rationale for, and objectives of, the Systematic Review clearly stated?

Partly

Is the statistical analysis and its interpretation appropriate?

Yes

If this is a Living Systematic Review, is the ‘living’ method appropriate and is the search schedule clearly defined and justified? (‘Living Systematic Review’ or a variation of this term should be included in the title.)

Not applicable

Are sufficient details of the methods and analysis provided to allow replication by others?

Yes

Are the conclusions drawn adequately supported by the results presented in the review?

Partly

Reviewer Expertise:

Social studies education, civics education, history and education, museum education

I confirm that I have read this submission and believe that I have an appropriate level of expertise to confirm that it is of an acceptable scientific standard.

# Reviewer report: Reviewer response for version 2

**DOI:** 10.5256/f1000research.176783.r360892

**Published:** 2025-02-07

**Authors:** Anissa Lestari Kadiyono

**Affiliations:** 1 Universitas Padjadjaran, Bandung, West Java, Indonesia

Abstract:

- In the abstract, the problems that occur still are not depicted so what is the problem with democratic values, and why through natural sciences education.

**Introduction:**

 Still, there is no improvement from this review:

- The research was opened by stating that “Colombia and the world have faced historical crises due to armed conflicts, development processes, and the influence of global ideologies. Events such as the Second World War, the Cold War, and the Latin American dictatorships stand out, whose effects have left a mark on generations’ thinking and behavior. However, despite their negative impact, these events are fundamental for developing a reflective, scientific, and proactive society. Hence, an urgent need arises to cultivate a culture of peace that encompasses various dimensions of social coexistence, environmental preservation, personal growth, and interactions between individuals and knowledge”. This is not clear to illustrate the problem of the importance of democratic values. Explain the context of the research and the importance of the problem so that democratic values need to be researched.
- Overall, natural science education has no basis in theory/ application. It is necessary to explain what is the content of this education both in content and in process and what kind of gap research this research can fill.

Are the rationale for, and objectives of, the Systematic Review clearly stated?

Partly

Is the statistical analysis and its interpretation appropriate?

Yes

If this is a Living Systematic Review, is the ‘living’ method appropriate and is the search schedule clearly defined and justified? (‘Living Systematic Review’ or a variation of this term should be included in the title.)

Yes

Are sufficient details of the methods and analysis provided to allow replication by others?

Yes

Are the conclusions drawn adequately supported by the results presented in the review?

Partly

Reviewer Expertise:

Psychology; Industrial & Organizational Psychology; Gender; Human Capital; Organizational Behavior

I confirm that I have read this submission and believe that I have an appropriate level of expertise to state that I do not consider it to be of an acceptable scientific standard, for reasons outlined above.

# Reviewer report: Reviewer response for version 1

**DOI:** 10.5256/f1000research.169050.r338091

**Published:** 2024-12-05

**Authors:** Anissa Lestari Kadiyono

**Affiliations:** 1 Universitas Padjadjaran, Bandung, West Java, Indonesia

**STUDY AIM:**

- This study examines how democratic values have been promoted through natural sciences education over the last 50 years, providing a comprehensive analysis based on a systematic review of relevant literature.
- However, the problems that occur are not depicted so what is the problem with democratic values and why through natural sciences education.

**Introduction:**

- The research was opened by giving a statement that “Colombia and the world have faced historical crises due to armed conflicts, development processes, and the influence of global ideologies. Events such as the Second World War, the Cold war, and the Latin American dictatorships stand out, whose effects have left a mark on generations’ thinking and behavior. However, despite their negative impact, these events are fundamental for developing a reflective, scientific, and proactive society. Hence, an urgent need arises to cultivate a culture of peace that encompasses various dimensions of social coexistence, environmental preservation, personal growth, and interactions between individuals and knowledge”. This is not clear to illustrate the problem of the importance of democratic values. Explain the context of the research and the importance of the problem so that democratic values need to be researched.
- Overall, natural science education has no basis in theory or application. It is necessary to explain what is the content of this education, both in content and in process, and what kind of gap research this research can fill.
- There is no relevant research and connection with previous research (novelty and merit)

**RESULTS:**

 In the result section, several important points have not been presented in the article, namely:

1. The explanation of the research results is based on the results of a systematic literature review and lacks an additional explanation of previous research and how these results are explained to the phenomena in the field.
2. This study only presents data with little information. There should be many things that can be elaborated on in this research.

**DISCUSSION:**

 In the discussion section, no in-depth elaboration of research results was found, especially regarding the discussion of the uniqueness of the results found in this study.

**CONCLUSION:**

 Improve the conclusion section to be more compact and summarize the research.

Are the rationale for, and objectives of, the Systematic Review clearly stated?

Partly

Is the statistical analysis and its interpretation appropriate?

Yes

If this is a Living Systematic Review, is the ‘living’ method appropriate and is the search schedule clearly defined and justified? (‘Living Systematic Review’ or a variation of this term should be included in the title.)

Yes

Are sufficient details of the methods and analysis provided to allow replication by others?

Yes

Are the conclusions drawn adequately supported by the results presented in the review?

Partly

Reviewer Expertise:

Psychology; Industrial & Organizational Psychology; Entrepreneurship; Gender; Human Capital; Organizational Behavior

I confirm that I have read this submission and believe that I have an appropriate level of expertise to state that I do not consider it to be of an acceptable scientific standard, for reasons outlined above.

# Response

**Published:** 2025-01-08

**Authors:** Ana Dolores Vargas Sánchez

**Affiliations:** Cundinamarca, Universidad de La Sabana, Chia, Cundinamarca, Colombia

We are grateful for the reviewer's comments and observations, as they have been extremely beneficial to our article.

 The subsequent elements have been modified:

- The introduction's final three paragraphs were examined. Details regarding the investigation's objective are provided.
- The introduction elaborated on and established the specific issue.
- The content of natural science education and the void that this research can address were clarified.
- The introduction was enhanced by the inclusion of references to the historical development of literature.
- The results were supplemented with relationships that were based on previous research, and the manner in which these relationships elucidated the phenomena in the field.
- An endeavor was made to establish a more direct connection between the results and the discussion.
- The conclusions section has been revised to be more succinct.

Thank you.

# Reviewer report: Reviewer response for version 1

**DOI:** 10.5256/f1000research.169050.r323025

**Published:** 2024-10-03

**Authors:** Genç Osman ilhan

**Affiliations:** 1 Yildiz Teknik Universitesi, Istanbul, İstanbul, Turkey

The article addresses the integration of the democratic values in natural science education in a well-organized document. The rationale and the objectives of the article are stated with references to government and union documents, which are among the strengths of the study. The rationale is supported with the UNESCO and EU documents; but the academic literature leading to the creation of this study is not explained through. The existing literature on the topic is recommended to be thoroughly presented and discussed in the "Introduction" part. 

 Another strength of the article is the construction and reporting of the methodology. The PRISMA methodology enabled the researcher to conduct the systemic review in a consistent and effective way. Also, the manuscript provides detailed information on the selection of the data and the analysis.

 The results part is enriched with sourceful data and visual tools such as figures. In the "discussion" part, the author(s) presented the existing literature on the topic. However, discussing the results of the study with the literature would be more convincing and effective. The results need to be matched with the references given in the manuscript and discussed through.

 In the conclusion part, the author refers to  Herzog and Lepenies without a reference number. It needs to be revised and corrected. 

 The article is well-designed and contributory to the field. However, the introduction and discussion parts need to be worked through. The discussions on the significant topics such as democracy, civic education and the others need to be discussed with references to the existing data rather than summarization

Are the rationale for, and objectives of, the Systematic Review clearly stated?

Partly

Is the statistical analysis and its interpretation appropriate?

Yes

If this is a Living Systematic Review, is the ‘living’ method appropriate and is the search schedule clearly defined and justified? (‘Living Systematic Review’ or a variation of this term should be included in the title.)

Not applicable

Are sufficient details of the methods and analysis provided to allow replication by others?

Yes

Are the conclusions drawn adequately supported by the results presented in the review?

Partly

Reviewer Expertise:

Social studies education, civics education, history and education, museum education

I confirm that I have read this submission and believe that I have an appropriate level of expertise to confirm that it is of an acceptable scientific standard, however I have significant reservations, as outlined above.

# Response

**Published:** 2025-01-08

**Authors:** Ana Dolores Vargas Sánchez

**Affiliations:** Cundinamarca, Universidad de La Sabana, Chia, Cundinamarca, Colombia

The reviewer's comments have been extremely beneficial in improving the content. We are grateful for their input.

 We changed as follows: 

- The introduction encompassed a more detailed examination of the more specific literature.
- Reviewed and changed, the discussion included writers to the outcomes.
- Reviewing and changing the conversation allowed the issues of democracy, civic education, and others more importance.
- The reference that shown discrepancies has been corrected.

Thank you.
